# Spatial synchronization codes from coupled rate-phase neurons

**DOI:** 10.1371/journal.pcbi.1006741

**Published:** 2019-01-25

**Authors:** Joseph D. Monaco, Rose M. De Guzman, Hugh T. Blair, Kechen Zhang

**Affiliations:** 1 Biomedical Engineering Department, Johns Hopkins University School of Medicine, Baltimore, MD, USA; 2 Psychology Department, University of California, Los Angeles, Los Angeles, CA, USA; University College London, UNITED KINGDOM

## Abstract

During spatial navigation, the frequency and timing of spikes from spatial neurons including place cells in hippocampus and grid cells in medial entorhinal cortex are temporally organized by continuous theta oscillations (6–11 Hz). The theta rhythm is regulated by subcortical structures including the medial septum, but it is unclear how spatial information from place cells may reciprocally organize subcortical theta-rhythmic activity. Here we recorded single-unit spiking from a constellation of subcortical and hippocampal sites to study spatial modulation of rhythmic spike timing in rats freely exploring an open environment. Our analysis revealed a novel class of neurons that we termed ‘phaser cells,’ characterized by a symmetric coupling between firing rate and spike theta-phase. Phaser cells encoded space by assigning distinct phases to allocentric isocontour levels of each cell’s spatial firing pattern. In our dataset, phaser cells were predominantly located in the lateral septum, but also the hippocampus, anteroventral thalamus, lateral hypothalamus, and nucleus accumbens. Unlike the unidirectional late-to-early phase precession of place cells, bidirectional phase modulation acted to return phaser cells to the same theta-phase along a given spatial isocontour, including cells that characteristically shifted to later phases at higher firing rates. Our dynamical models of intrinsic theta-bursting neurons demonstrated that experience-independent temporal coding mechanisms can qualitatively explain (1) the spatial rate-phase relationships of phaser cells and (2) the observed temporal segregation of phaser cells according to phase-shift direction. In open-field phaser cell simulations, competitive learning embedded phase-code entrainment maps into the weights of downstream targets, including path integration networks. Bayesian phase decoding revealed error correction capable of resetting path integration at subsecond timescales. Our findings suggest that phaser cells may instantiate a subcortical theta-rhythmic loop of spatial feedback. We outline a framework in which location-dependent synchrony reconciles internal idiothetic processes with the allothetic reference points of sensory experience.

## Author summary

Spatial cognition in mammals depends on position-related activity in the hippocampus and entorhinal cortex. Hippocampal place cells and entorhinal grid cells carry distinct maps as rodents move around. The grid cell map is thought to measure angles and distances from previous locations using path integration, a strategy of internally tracking self motion. However, path integration accumulates errors and must be ‘reset’ by external sensory cues. Allowing rats to explore an open arena, we recorded spiking neurons from areas interconnected with the entorhinal cortex, including subcortical structures and the hippocampus. Many of these subcortical regions help coordinate the hippocampal theta rhythm. Thus, we looked for spatial information in theta-rhythmic spiking and discovered ‘phaser cells’ in the lateral septum, which receives dense hippocampal input. Phaser cells encoded the rat’s position by shifting spike timing in symmetry with spatial changes in firing rate. We theorized that symmetric rate-phase coupling allows downstream networks to flexibly learn spatial patterns of synchrony. Using dynamical models and simulations, we showed that phaser cells may collectively transmit a fast, oscillatory reset signal. Our findings develop a new perspective on the temporal coding of space that may help disentangle competing models of path integration and cross-species differences in navigation.

## Introduction

A prominent temporal code of neural activity [1–3] is the phase precession of rodent place cell and grid cell activity relative to the septal-hippocampal theta rhythm (6–11 Hz) [4, 5], in which firing begins late in the theta cycle and advances to earlier phases as the animal moves across a spatial firing field. Theta-phase precession is strictly unidirectional, which ensures that phase unambiguously encodes the distance traveled through a place field [6]. This unidirectionality may follow from mechanisms such as neuronal adaptation that halts firing before the peak of dendritic excitation [7], place-cell network plasticity that learns an asymmetric ramp of depolarizing input through experience [8], or temporal interference between a somatic theta oscillation and a speed-tuned [4, 9] or spatial [7, 10–12] dendritic oscillation. In open-field foraging, these mechanisms may lock the phase-distance code of phase precession to trajectory details (that is, the speed, running direction, and path) of individual passes through a spatial firing field [13, 14], thus preventing a direct mapping of phase to spatial locations. It is unclear whether phase codes with different properties (for example, bidirectionality, spatial symmetry, or trajectory independence) operate in other brain areas to process spatial information.

Temporal interference models theorized that multiple velocity-controlled oscillators (VCOs) [15, 16] perform path integration to collectively synthesize the hexagonally periodic spatial firing of grid cells [17]. Electrotonic soma-dendrite coupling ruled out dendritic implementations of VCOs [18], leading to models of neuronal oscillators that project path-integrating phase codes to the grid cell network [19–21]. Experimental evidence for neuronal VCOs includes our previous report of thalamic theta-bursting neurons with the theoretically required burst-frequency tuning of direction [22] and observations of full phase precession at the periphery of grid cell fields as predicted by temporal interference but not continuous attractor networks or ramp depolarization models [14, 23–25]. Organizing VCOs into ring attractor networks provides some internal stability [26, 27], but biological variance in spike timing and local theta cycle periods limits the temporal precision of VCO phase computations [28, 29]. Likewise, continuous attractor models of grid-cell path integration accumulate position errors, even before considering sources of biological variance. In open environments that allow rotations, and particularly at low speeds, bounded network topologies cause error-inducing ‘ripples’ that perturb an otherwise flat energy landscape [30].

To counter the accumulation of position errors, path integrators must reset to the current position based on environmental cues [31, 32]. Models combining the continuous attractor and VCO frameworks have proposed resetting VCOs via descending grid cell feedback [27, 33, 34]. However, for mice in complete darkness, grid cell patterns are rapidly disrupted [35] while path integration is sufficiently preserved to maintain a global heading angle [36]. Thus, grid cell networks in different species may not have the spatial stability to support a feedback role (as in the combined attractor/oscillator models) and may not directly compute the spatial vector maintained by path integration (as in continuous attractor models).

Subcortical targets of the hippocampal formation, typically studied as regulators of the theta rhythm (cf. [37, 38]), may additionally contribute to neural computations of space. In rats, the lateral septum (LS), but not the medial septum, has revealed spatial modulation of firing rates in open environments [39, 40] that diverged with respect to hippocampal remapping over time [41]. However, LS neurons have also been reported to carry a phase code for one-dimensional (1D) tracks that precisely reflected hippocampal phase precession [42]. The degree to which LS or other spatially-modulated subcortical neurons are computationally dependent on hippocampal activity is unclear, especially in open two-dimensional (2D) environments.

In this study, we asked two questions: (1) Can spatial theta-phase codes be found in subcortical theta-rhythmic structures? (2) What computational function might such phase codes serve in downstream circuits related to spatial cognition? Our approach integrated, respectively, single-unit recordings in rats during open-field foraging, and computational modeling of spatial phase-coding networks and their downstream targets. We found a class of LS and hippocampal neurons with 2D spatial phase codes for which we analyzed the relationship between rate and phase, stability of rate and phase coding, temporal organization by theta, spatial firing patterns, and spatial vs. trajectory-related selectivity. Our analysis was consistent with an absolute, allocentric representation of space, thus we studied models of temporal coding mechanisms distinct from those hypothesized for the relative, field-centered representation of hippocampal phase precession. We suggest the theory that intrinsic neuronal and network processing of convergent hippocampal inputs form an independent and collective encoding of the animal’s current (not prospective) position. This spatial transformation may enable rapid and flexible phase-resetting of path integration.

## Results

We will first describe recordings of subcortical and hippocampal theta-modulated neurons in freely behaving rats. By setting criteria for spatial phase coding, we analyzed a subset of these neurons that we termed ‘phaser cells’ to reveal how spatial information was carried in the phase alignment of firing with the hippocampal theta oscillation observed in local field potentials (LFPs). We posit a theoretical account of the relationship between firing rate, shifts in spike phase, and ongoing theta oscillations that is supported by generalized linear models (GLMs) trained across a spatial partition of the recording arena. Lastly, we demonstrate models of intrinsic theta-bursting and spike synchronization in both artificial 1D and realistic 2D simulations of phaser cells that collectively corrected phase-position errors in downstream path-integration networks.

### Modulation of firing rate and phase by position

We obtained tetrode recordings from 8 rats as they foraged in an 80-cm cylindrical arena during sessions lasting an average of 2.1 hours. Long sessions helped to ensure sufficient sampling of phase differences across the environment. Hippocampal LFP signals were recorded from an electrode located in the hippocampal stratum oriens, referenced to animal ground. Across 110 sessions, LFPs were collected concurrently with 1,073 single-unit recordings (we use ‘recording’ to refer to a unit’s data from one session) of 671 uniquely identified neurons (some of which were observed in multiple recordings) from sites including the LS and medial septum, hippocampus, thalamus, midbrain, and other subcortical areas (Table 1; Methods).

**Table 1:** Identified cell counts from single-unit recordings by brain area and spatial phase-coding subtype.

Recording Area	Negative	Positive	Mixed	None	Total
Lateral septum	31 (9.7%)	17 (5.3%)	2 (0.6%)	287 (84.4%)	321
Medial septum	–	–	–	16 (100.0%)	16
Hippocampus	11 (12.4%)	4 (4.5%)	–	74 (83.1%)	89
Thalamus	1 (2.2%)	–	–	45 (97.8%)	46
Midbrain	1 (0.7%)	–	–	134 (99.3%)	135
Other	1 (1.6%)	–	1 (1.6%)	62 (96.8%)	64
Total	45 (6.7%)	21 (3.1%)	3 (0.4%)	602 (89.7%)	671

Columns: ‘Negative’/‘Positive’, cells with at least one negative/positive phaser-classified recording and none of the other subtype; ‘Mixed’, cells with at least one negative and at least one positive phaser-classified recording; ‘None’, cells with no phaser-classified recordings.

**Fig 1:**
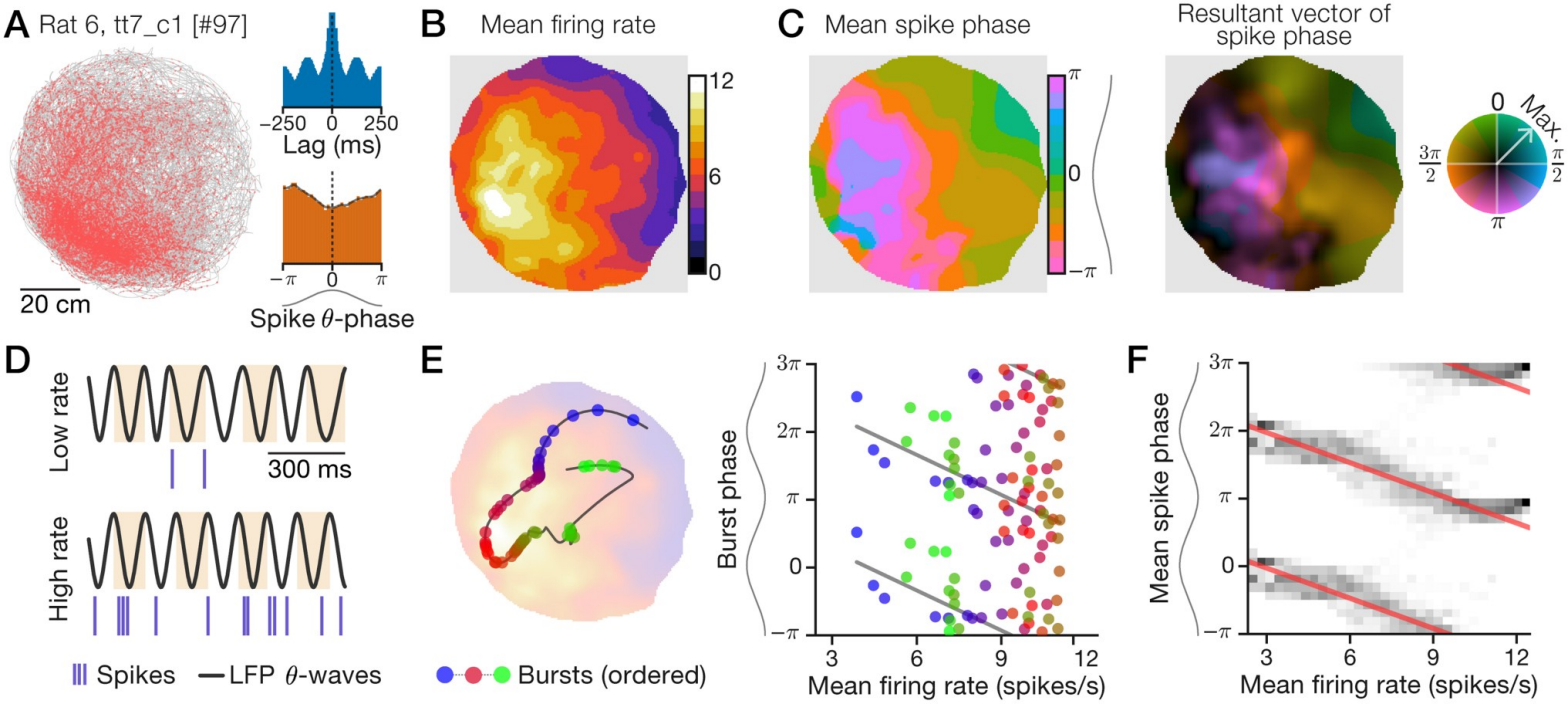
An example LS neuron with spatially correlated rate and phase. Recording data for a 2.2-h session in an 80-cm diameter arena. Sinusoids aligned to phase axes indicate theta waves, with peaks oriented to the top (horizontal axes) or left (vertical axes). (A) Spike-trajectory plot. Red dots: animal position at time of spike; gray line: trajectory. Inset: spike train autocorrelogram (top) and spike theta-phase distribution (bottom). (B+C) Spatial maps computed with an adaptive Gaussian kernel (Methods). (B) Firing ratemap. (C) Spike mean-phase map (left) and phase-vector map (right) with normalized MVL indicated by color saturation (color wheel; Methods). (D) Example 1-s traces of hippocampal LFP theta waves and spikes during periods of low (top) or high (bottom) firing rate. Highlights show theta cycles. (E) Example 15-s trajectory segment (line) showing bursts (circles) emitted as the rat traveled from a low-rate region to a high-rate location (blue-to-red bursts) and back to a low-rate region (red-to-green bursts; left). Likewise, plotted against firing rate, burst phase first advanced (blue-to-red bursts) and then delayed (red-to-green bursts; right). Left background: ratemap from (B). (F) Mean-phase (C) distributions (grayscale) conditioned on mean rate (B). Red lines: circular-linear regressions; multiple theta cycles shown (*y*-axis) for clarity.

In some recordings, units exhibited spatial tuning of firing rate as well as spatial tuning of spike phase with respect to the LFP theta oscillation. Fig 1 shows one such cell from LS that fired preferentially in the west/southwest of the arena (Fig 1A) and was moderately theta-rhythmic (index: 0.392; Fig 1A, inset, top; Methods) and theta-modulated (index: 0.288; Fig 1A, inset, bottom; Methods). Across space, the cell’s mean firing rate (‘ratemap’; Fig 1B; Methods) revealed a single-peaked firing field that broadly covered much of the arena. Surprisingly, the spatial distribution of the mean theta-phase of spikes (‘mean-phase map’; Fig 1C, left; Methods) varied in a pattern of spatial modulation that qualitatively matched the ratemap in Fig 1B. The cell fired at LFP theta peaks (0 radians) in locations corresponding to low firing rates (Fig 1C, left, green regions) and during example low-firing-rate time intervals (Fig 1D, top). Conversely, the cell fired near LFP theta troughs (−π or π radians) in locations corresponding to high firing rates (Fig 1C, left, pink regions) and example high-firing-rate intervals (Fig 1D, bottom). To quantify phase reliability during a recording, we computed at every location the mean resultant vector length (MVL) of spike phase, which varies from 0 (uniformly random) to 1 (perfectly reliable). Thus, we display the full effect of spatial modulation on spike phase with a ‘phase-vector map’ (or simply ‘phase map’) where mean phase is indicated by color hue (as in Fig 1C, left) and maximum-normalized MVL by color saturation (Fig 1C, right; Methods). The example cell had typical phase MVL around 0.2 except for a high-variance region along the westward wall (Fig 1C, right, dark pixels) and a high-reliability region >0.3 near the center of the arena (Fig 1C, right, bright pixels).

### Quantifying selection criteria for spatial phase coding

To study the characteristic phase relationships in our data, we examined spiking activity over individual traversals of the arena and whole-session spatial maps. A 15-s trajectory segment illustrates a series of bursts emitted by the example LS neuron (Fig 1E, left). The cell initially burst around theta peak in a low-rate region in the northeast of the arena, precessed to earlier phases in the high-rate region as the animal moved to the southwest, and then shifted back to later phases when the animal returned to a low-rate region (Fig 1E). Burst phase during this short trajectory was noisy, but the activity symmetrically followed the rate-phase regression line in both directions (Fig 1E, right), corresponding first to phase advance and then to phase delay. To measure this phase modulation over the 2.2-h session, we regressed the mean-phase map (Fig 1C, left) onto the ratemap (Fig 1B), revealing a negatively sloped rate-phase relationship (circular-linear correlation: *n* = 3,190 map pixels, estimated $\hat{r}=-\text{0.836}$, $\hat{p}\approx 0$; Methods) around which the cell’s spatial data was narrowly distributed (Fig 1F). For this cell, spike phase was symmetrically and bidirectionally coupled to firing rate over multiple timescales.

By inspecting our dataset for this phenomenon, we defined ‘phaser cells’ as neurons whose spike phase coded for position and was strongly coupled to firing rate. To classify phaser cell recordings, we imposed criteria on three measures of phase, rate, and space (Methods): (1) Spatial phase information *I*_phase_ quantified the spatial content of spike alignment to LFP theta oscillations as the Shannon mutual information between spike phase and position; (2) Total phase shift captured the depth of phase modulation as the regressed phase difference from the minimum to maximum rate; (3) The rate-phase correlation indicated the strength of rate-phase coupling based on a recording’s ratemap and mean-phase map.

To determine the criteria, we asked how recordings that carried spatial information in spike theta-phase differed from others. Significant phase-coding recordings (*I*_phase_ shuffled phase test, *p* < 0.02; *n* = 156 cells; S1 Fig, panel D) exhibited less variable theta-burst frequency (variance ratio, 0.624; *I*_phase_-significance bootstrap test, *p* = 0.001; Methods) than non-significant recordings (*n* = 570 cells; S1 Fig, panel B), suggesting that phase-coding cells were more reliably periodic. Furthermore, significant phase-coding recordings exhibited more variable rate-phase correlation coefficients (variance ratio, 3.87; *p* = 0.001) and more broadly distributed total phase shifts (interquartile range ratio, 1.96; *p* = 0.001) than non-significant recordings (S1 Fig, panel E). Thus, we classified phaser cell recordings as unit-session data that met each of several criteria:

1. Spatial phase information *I*_phase_ must be significant (*p* < 0.02) and ≥ 0.1 bits;
2. The magnitude of the total phase shift must be ≥ *π*/4 radians;
3. The estimated rate-phase correlation coefficient must be significant ($\hat{p}<0.02$) with absolute value $|\hat{r}|\geq 0.2$; and
4. The maximal firing rate of the ratemap must be ≥ 3.5 spikes/s.

The fourth criterion ensured sufficient levels of spatial activation, at least one spike every other theta cycle, to convey rate and phase relationships. A total of 101 recordings from 5 rats satisfied the phaser cell criteria. Phaser cell recordings revealed moderate firing rates, corresponding to 1 or 2 spikes per theta cycle in preferred regions, and similar theta rhythmicity to other significant phase-coding recordings (S2 Fig, panel A). By analyzing which recordings followed the same neuron across multiple sessions (Methods), we determined that 69 unique phaser cells were observed by the 101 recordings: 50 phaser cells were located in the lateral septum, 15 in the hippocampal formation, and 4 in other subcortical structures (Table 1).

### Mapping high-rate regions with timing advance or delay

**Fig 2:**
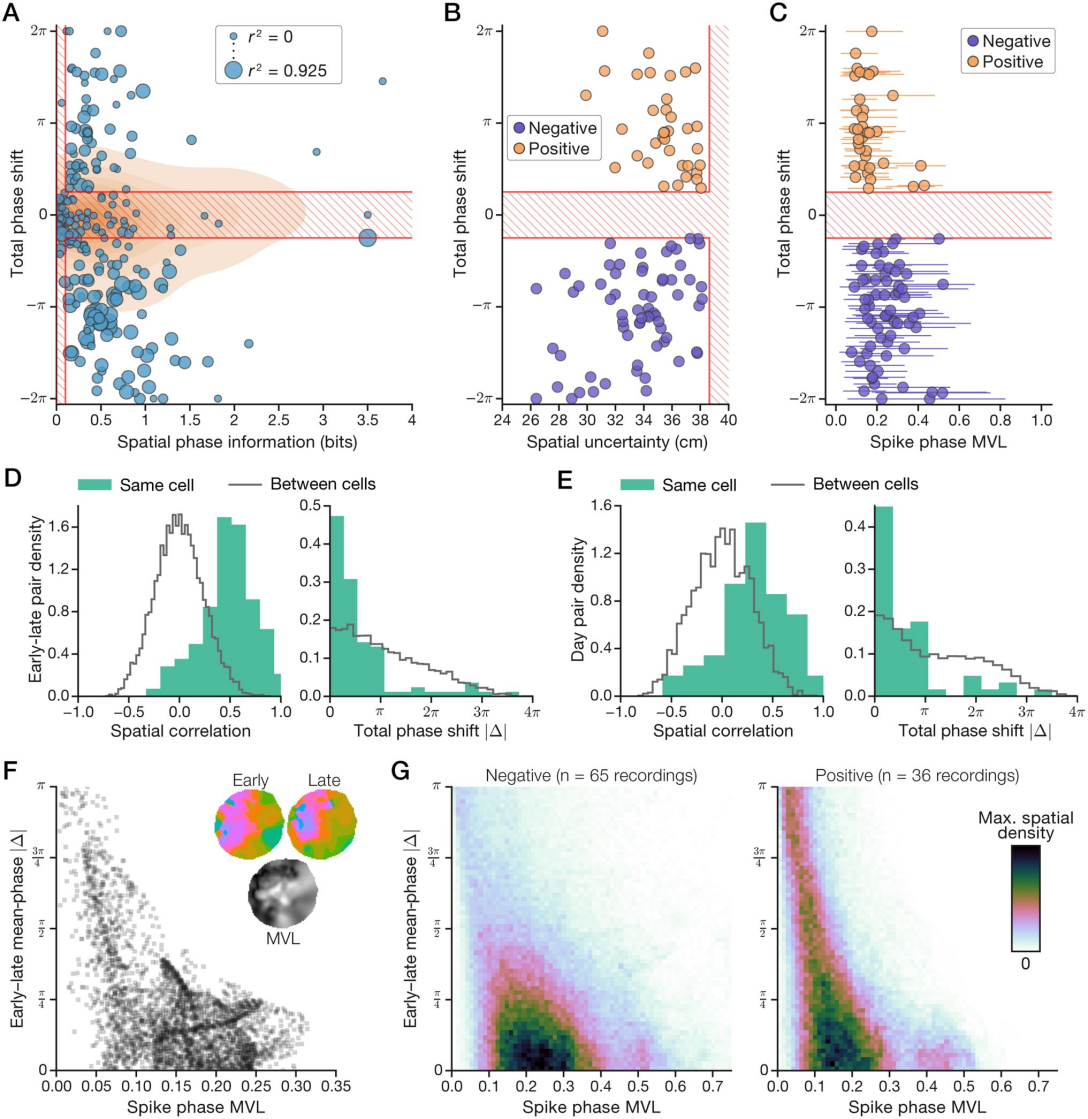
Phaser cells encode space with positive or negative phase shifts. (A) Selection of phase-coding recordings based on spatial phase information (*I*_phase_, *x*-axis), total phase shift (*y*-axis), and rate-phase coupling strength (circle diameter). Phaser cell recordings were divided into ‘negative’/‘positive’ subtypes according to the bottom-right/top-right regions selected by the criteria. Circles: significant *I*_phase_ recordings; contours: kernel density-estimate of non-significant recordings; red hatch lines: region excluded by the first two phaser cell criteria (see numbered listing of criteria above in Results). (B) Spatial uncertainty is related to the magnitude of phase shift for negative and positive phaser cell recordings. (C) Spatial distributions of mean resultant vector length (MVL) across phase maps (mean ± 90% empirical c.i.). (D+E) Pair-wise comparisons of early vs. late (<1 h) session activity (D) or between days (E). Within-cell spatial correlations were higher (left) and absolute changes in total phase shift were lower (right) than baseline comparisons between cells. Histograms: normalized by pair count, bin size from the Freedman-Diaconis rule. (F+G) Spatial comparison of MVL (*x*-axis) and within-session change in the phase code (*y*-axis) at every location in the phase map. (F) Example LS cell from Fig 1. Inset: mean-phase maps (top) and whole-session MVL (bottom; black, 0; white, maximum MVL). (G) Average density of all negative (left) and positive (right) phaser cell recordings.

The validity of the above criteria for phaser cells depended on whether they selected a meaningful subset of our data. Fig 2A visualizes the measures tested by the first two criteria (*I*_phase_ and total phase shift) with respect to their thresholds; the third measure (rate-phase coupling strength) is indicated by the size of the plot markers. In Fig 2A, significant phase-coding recordings (*n* = 233) are shown with individual data points, the distribution of non-significant recordings (*n* = 840) is represented by contours in the background, and phaser cell criteria (1) and (2) above are overlaid as red lines that cross out the region excluded by the criteria. Non-significant recordings (Fig 2A, contours) displayed a wide range of *I*_phase_ values that failed to achieve statistical significance (S1 Fig, panel D) and no relationship with total phase shifts that were narrowly distributed around zero (S1 Fig, panel E, right). However, significant phase-coding recordings (Fig 2A, circles) fell into roughly three clusters: (1) low *I*_phase_, total phase shift near zero, and minimal rate-phase coupling; (2) moderate *I*_phase_, large positive phase shifts, and moderate coupling; (3) high *I*_phase_, large negative phase shifts, and strong coupling. The first cluster was excluded, and the latter two clusters were selected as phaser cell recordings. Due to the striking division of the direction of phase shifts between the selected clusters, we labeled them as ‘positive’ and ‘negative’ subtypes. That is, negative phaser cells advanced to earlier phases, like hippocampal phase precession, and positive phaser cells delayed to later phases, unlike previously described spatial phase codes.

To verify that differences in the direction of phase shifts were not artifacts of the recording configuration, we inspected our dataset for colocation, stability, and simultaneous observation of the two subtypes. Phaser cells were predominantly recorded from LS (Table 1; S3 Fig, panel A). Two-thirds of phaser cells (48/69) were negative and one-third (24/69) were positive. For 19 phaser cells with multiple recordings, all but 3 preserved the sign of phase shift across their phaser-classified recordings (S2 Fig, panel B, right). In some cases, negative and positive phaser cells were recorded simultaneously against the same LFP reference electrode and/or observed on the same tetrode. These observations, together with the fact that the LFP signal was always recorded from the hippocampal stratum oriens, indicate that the direction of rate-phase coupling was a stable property of individual phaser cells and not an artifact of variations in LFP signal polarity.

### Accuracy and reliability of the phaser cell code

To quantify phaser cell accuracy and reliability, we examined, respectively, a measure of spatial uncertainty and the spatial distribution of spike-phase MVL. We computed spatial uncertainty as $R/\sqrt{2^{I_{\text{phase}}}}$ for arena radius *R* = 40 cm. Increasing magnitude of total phase shift was associated with lower spatial uncertainty for negative (*n* = 65 recordings; mean ± s.e.m., 33.5 ± 0.378 cm; linear regression, *r* = 0.363, *p* = 0.00292) and positive (*n* = 36; 35.4 ± 0.349 cm; *r* = −0.441, *p* = 0.00707) phaser cells (Fig 2B). Across spatial locations, MVL was distributed from nearly zero up to a typical maximum value of 0.414 (median, *n* = 101 recordings; Fig 2C). In order to statistically test for differences between subtypes, we averaged values across recordings for unique cells with multiple recordings. Negative phaser cells demonstrated both lower spatial uncertainty (*n* = 48/24 negative/positive cells; *post hoc* Welch’s *t* = −2.32, *p* = 0.0236) and higher phase-code reliability (mean MVL; *t* = 2.68, *p* = 0.010) than positive phaser cells. Thus, phaser cells exhibited spatial accuracy on the order of body length based on a reliable mapping of spike phase to position in certain locations.

### Stability of spatial modulation and phase coding

If phaser cells contribute to navigation or other spatial functions, then they must stably reflect a given context or environment. Cell-specific spatial modulation and rate-phase coupling should be preserved over both long experiences and multiple days. To analyze spatial stability of phase coding in phaser cells, we compared early vs. late portions (<1 h) of each recording to a baseline of pair-wise measurements between different cells (Methods). For spatial stability, the distributions of spatial correlations between ratemaps revealed significant similarity above baseline across the multiple-hour recording sessions (median, 0.502; within-cell (*n* = 101) vs. between-cell (*n* = 9, 986) early-late pairs; Kolmogorov-Smirnov *D* = 0.694, *p* = 2.07e−43; Fig 2D, left). For phase-coding stability, changes in total phase shift were distributed narrowly around zero, significantly lower than baseline (1.07 radians; *D* = 0.371, *p* = 1.00e−12; Fig 2D, right). Likewise, for the 19 phaser cells with multiple recordings, spatial correlations between different recording days were significantly higher than baseline (0.345; within-cell (*n* = 57) vs. across-cell (*n* = 4, 986) day pairs; *D* = 0.431, *p* = 7.52e−10; Fig 2E, left) and changes in total phase shift were distributed close to zero, significantly lower than baseline (1.30 radians; *D* = 0.399, *p* = 1.66e−8; Fig 2E, right). Further, all but 3 of these phaser cells maintained similar *I*_phase_ values and total phase shifts across days (S2 Fig, panel B), suggesting a global stability of the phase code beyond the pair-wise stability implied by Fig 2E.

The stability of *I*_phase_ and total phase shift is necessary for phase-code stability, but those are spatially averaged measurements and relative phase shifts remain constant even if phase-code angles systematically drifted. Thus, we addressed the relationship between specific locations and the magnitude of changes in mean-phase angles. We calculated absolute phase differences between the early and late mean-phase maps from the analyses in Fig 2D. To relate these phase differences to spatial variation of phase reliability (Fig 2C), we display them according to spike-phase MVL. Low/high MVL locations would be expected to show larger/smaller phase differences over time. Fig 2F shows MVL and absolute early-late phase differences for the LS cell from Fig 1; the wedge shape reflects the expected relationship, but the placement of the bulk of the data distribution revealed that typical MVL values coincided with phase differences of <π/4 radians (that is, 1/8th of a theta cycle or ∼17 ms). Averaging across phaser cell recordings revealed a similar pattern in which the region of highest spatial density corresponded to absolute phase-code changes of <1/8th of a theta cycle (Fig 2G). As in Fig 2B+2C, positive phaser cells demonstrated weaker phase-coding than negative phaser cells, as shown by the relatively higher density of the ‘tail’ leading up to maximal phase difference (|Δ| = π) at low MVL (Fig 2G, right). Thus, phase reliability (Fig 2C) implied location-dependent phase-code stability over multiple hours (Fig 2G). The spatial and phase-coding stability of phaser cells across hours and days was consistent with functional contributions to the spatial computations of the hippocampal formation.

### Experience-independent phase coding of spatial isocontours

**Fig 3:**
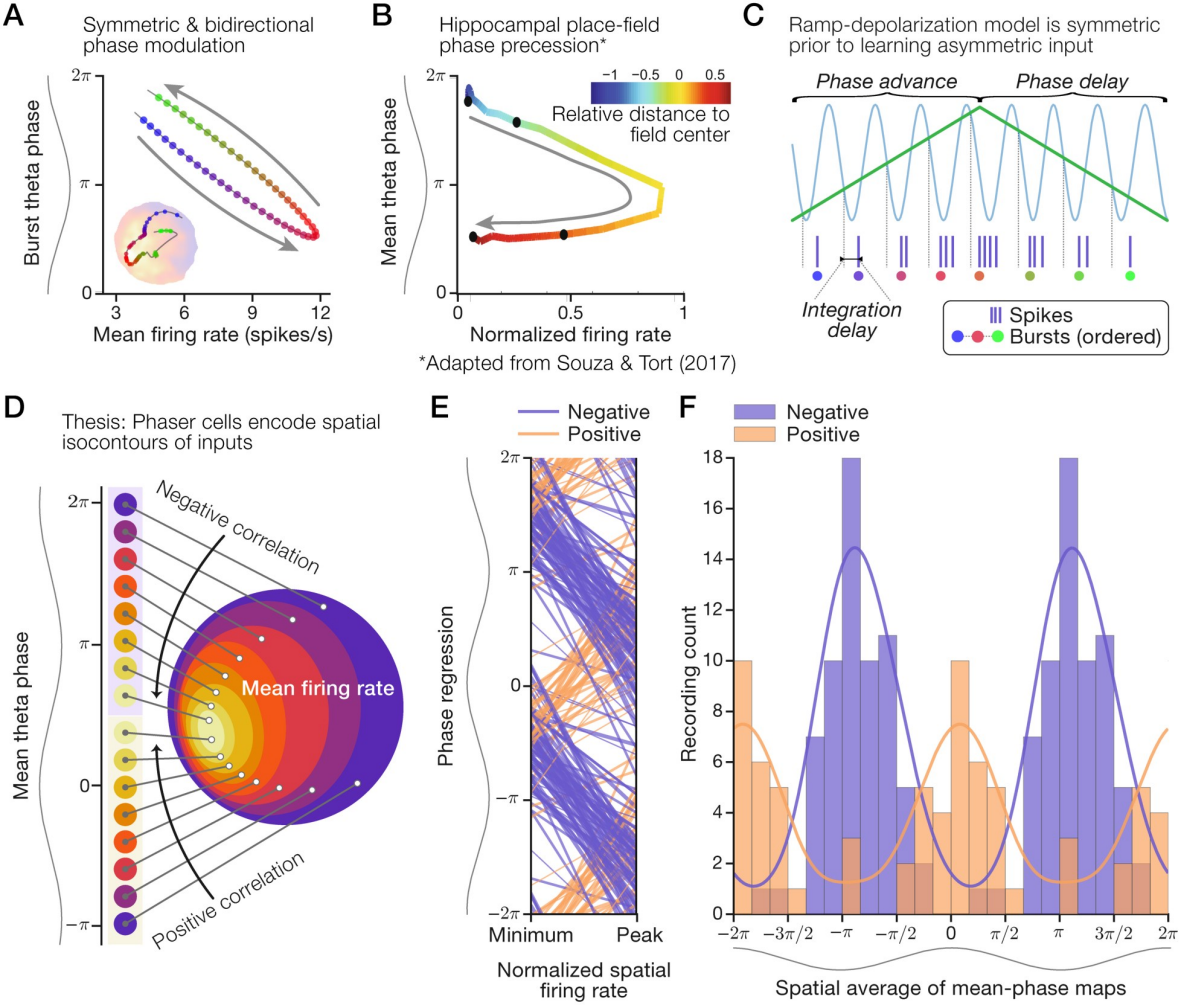
Mechanisms and temporal organization of the phaser cell code. Our thesis is that phaser cell activity is distinct from hippocampal phase precession and encodes spatial isocontours, not specific locations. (A) Schematic of symmetric rate-phase coupling (cf. Fig 1E, right) that deflects in one direction and then retraces in the opposite direction as the animal moves through a high-activity region. Inset from Fig 1E for illustration. (B) Mean rate-phase relationship across normalized traversals of 1,071 place fields from Souza & Tort (2017) [43]. Arrow: unidirectionality of phase precession. (C) Schematic of ramp-depolarization model with symmetric inputs, as is the case prior to learning [8]. Sinusoid: theta inhibition; green line: depolarizing input. (D) Schematic of a spatial phase code modeled on the LS cell in Fig 1 in which theta phase (left) maps to an isocontour level of underlying spatial inputs reflected by mean firing rate (right). (E+F) Negative and positive phaser cell recordings were segregated by theta phase. Multiple theta cycles shown for clarity. (E) Rate-phase regressions across normalized mean firing-rates. Line width: thin, |*r*| < 1/3; medium, 1/3 ≤ |*r*| < 2/3; thick, |*r*| > 2/3. (F) Distributions of typical spike theta-phases computed as spatial averages. Histograms: positive composited over negative; lines: density estimates using a circular π/4 bandwidth Gaussian kernel. Panel (B) was adapted from figure 5B of Souza & Tort (2017) [43] as permitted by the CC-BY 4.0 International License (creativecommons.org/licenses/by/4.0/).

We asked what theoretical mechanism could support our observations of the spatial phase code carried by phaser cells. We considered the crucial feature that spatial data points, such as the conditional spike-phase distributions in Fig 1F, were tightly coupled to the rate-phase regression. Strong rate-phase coupling suggested that the rate-phase relationship was maintained across spatial locations and that rate and phase did not systematically diverge over short or long timescales. We surmised that, on average, rate and phase deflected together on approaches to a preferred location (that is, a high mean firing-rate region), and then symmetrically retraced those deflections on leaving the preferred location (Fig 3A). Thus, we theorized that the phaser cell code was a spatially homogeneous coupling of rate and phase that was symmetric and, because they deflect and retrace, bidirectional.

In contrast, Souza & Tort (2017) [43] examined hippocampal place-cell theta-phase at low firing rates and revealed a distinct angle-shaped rate-phase relationship across place fields. The resulting curve (adapted in Fig 3B) reflects the combination of two effects that progress from entry to exit of hippocampal place fields: (1) the strict unidirectionality of spike theta-phase precession [4], and (2) the single-peaked rise and fall of firing rate, which may be symmetric or skewed with respect to the field center [12, 44]. To reconcile these differences, we suggest that symmetric, bidirectional phaser cell coding (Fig 3A) and asymmetric, unidirectional hippocampal phase precession (Fig 3B) reflect experience-independent vs. experience-dependent models of temporal coding, respectively. Mehta et al. (2002) [8] proposed that theta-rhythmic inhibition combines with spatially asymmetric input learned from the place-cell network to monotonically shift spike phase across place fields. However, absent learning, that mechanism generates a symmetric rate-phase relationship mediated by the rise and fall of external input (Fig 3C). Thus, theta-rhythmic inhibition combined with depolarization by external inputs may explain the rate-phase relationship of negative phaser cells (Figs 1F and 3A). As noted in Mehta et al. (2002) [8], coupling phase to rate precludes a precise mapping between phase and specific locations within a place field. Instead, a rate-coupled phase signal in a 2D environment is restricted to encoding isocontours of the depolarizing spatial input (Fig 3D; Discussion).

### Temporal segregation by direction of rate-phase coupling

Our observations of positive phaser cells, which modulated timing in the opposite direction to negative phaser cells, presented a conundrum. In models described below, we suggest a network mechanism to account for this difference, but the key prediction is that positive modulation requires theta-rhythmic excitation instead of inhibition. A consequence of theta excitation is that positive cells would fire at theta peak (0 radians) at low firing rates, and then delay to later phases at higher rates. Negative phaser cells based on a symmetric ramp mechanism (Fig 3C) would fire following the minimal inhibition of the theta trough (−π or π radians) at low firing rates, and then advance to earlier phases at higher rates. This distinction implies a temporal segregation of phaser cell activity. To assess this temporal organization, we show rate-phase regressions for every phaser cell recording according to subtype (Fig 3E). Negative and positive phaser cells fired during the rising phase [−π, 0] at low firing rates, and, with increasing firing rate, followed opposing paths to the falling phase [0, π], thus complementarily spanning the theta cycle (Fig 3E). Positive phaser cell activity clustered before theta peak at low rates (Fig 3E) as predicted by theta excitation and a high threshold. Distributions of typical spike phases, computed as the spatial average of mean-phase maps to avoid the sampling biases of time averages, show that the subtypes were segregated by theta phase: negative/positive phaser cells typically fired at theta trough/peak (Fig 3F). Thus, temporal segregation by subtype may reflect underlying differences in theta drive.

### Patterns of spatial modulation in phaser cells

**Fig 4:**
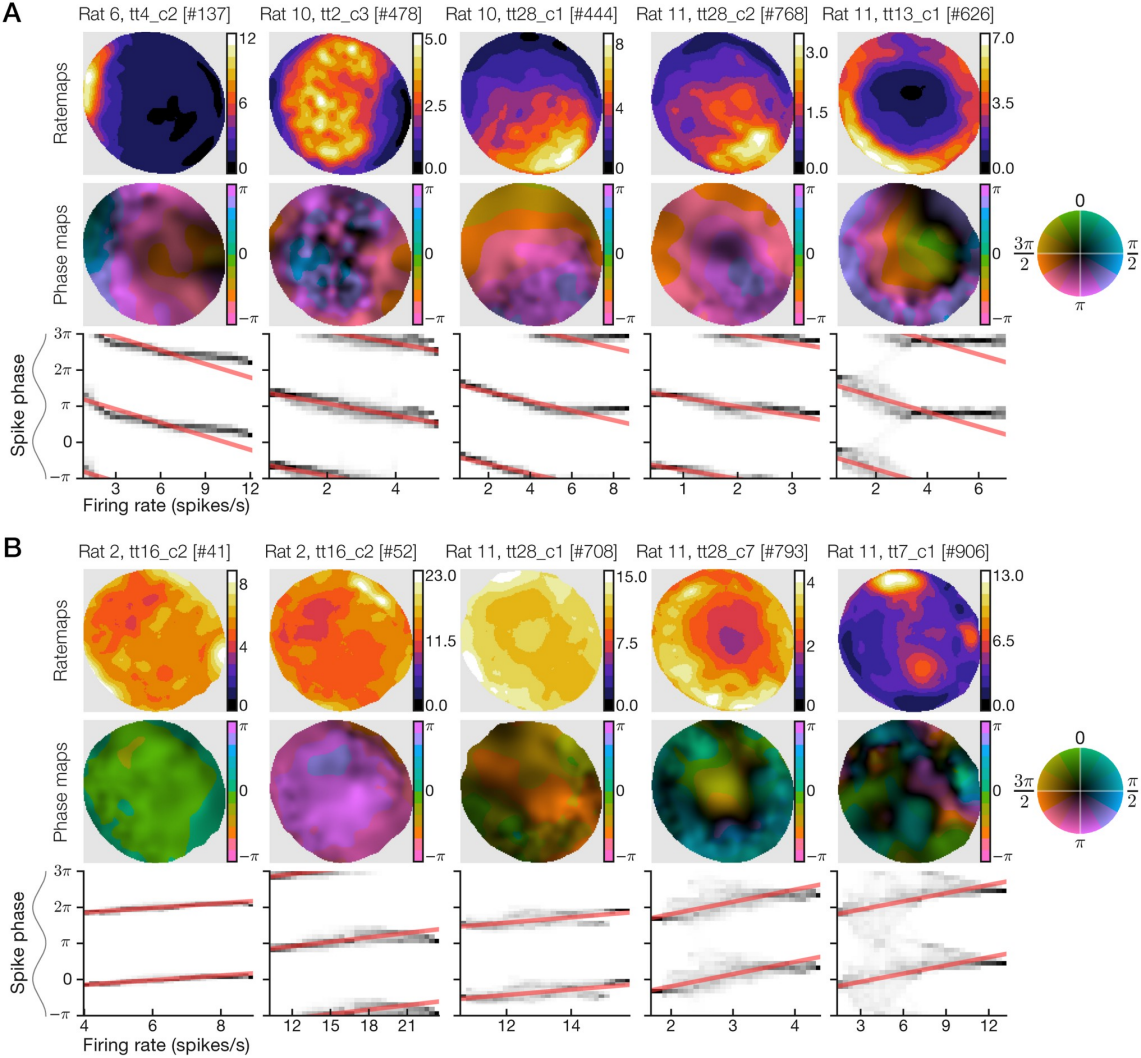
Example phaser cells illustrate the diversity of spatial phase codes. For example recordings of negative (A) and positive (B) phaser cells, we show the ratemap (top), phase-vector map (middle), and conditional spike-phase distribution with rate-phase regression lines (bottom, as in Fig 1F). Maximal firing rates (top rows, color bar axes) were consistent with the moderate range of phaser cell firing rates (S2 Fig, panel A, left). Negative phaser cells demonstrated visibly stronger spatial modulation and rate-phase coupling compared to positive phaser cells, consistent with analyses of spatial uncertainty (Fig 2B), phase reliability (Fig 2C), and location-specific phase-code stability (Fig 2G).

Negative phaser cell ratemaps revealed diverse spatial representations including place-like fields, broad gradient-like fields, and boundary (including on/off) responses along the arena wall (Fig 4A; recordings #444 and #768 produced remarkably similar rate and phase maps from different rats). Maximal firing rates (Fig 4A, top) corresponded to pre-theta-trough timing (Fig 4A, middle, blue/pink). Conditional spike-phase distributions (Fig 4A, bottom) revealed a tendency for phase modulation to halt after approximately one-half theta cycle, perhaps indicating a minimum latency to spike following theta-peak inhibition; this nonlinearity means that some rate-phase regression lines (Fig 3E) overestimated the total phase shifts. Positive phaser cells likewise showed diverse spatial modulation, but the responses were more subtle, involving higher baseline firing rates and heterogeneous compositions of boundary-like and place-like selectivity (Fig 4B, top). Maximal firing rates typically mapped to post-theta-peak timing (Fig 4B, middle, green/blue) and the rate-phase relationships were weaker (*n* = 24 cells; median, rate-phase correlation $\hat{r}=0.42$; Fig 4B, bottom) than those of negative phaser cells (*n* = 48; $\hat{r}=-0.54$; Fig 4A, bottom; absolute values, *post hoc* Welch’s *t* = 2.053, *p* = 0.0442). Thus, subtype differences in patterns of spatial modulation reinforced our analysis showing higher spatial uncertainty and weaker phase stability in positive phaser cell recordings (Fig 2B+2C and 2G).

To quantify spatial modulation, we calculated spatial rate information *I*_rate_ using a standard measure of position coding in place cells [45] and determined its statistical significance in phaser cell recordings with a spike-train shift test (criterion *p* < 0.02; Methods); 47/48 negative and 24/24 positive phaser cells attained significance. As expected from prior analyses, negative phaser cell spikes carried significantly higher *I*_rate_ (*n* = 47 significant cells, *p* < 0.02; 0.381 ± 0.06 bits/spike, mean ± s.e.m.) than positive phaser cell spikes (*n* = 24, 0.111 ± 0.048; log values, *post hoc* Welch’s *t* = −3.92, *p* = 0.0002). The least-squares optimized slope between *I*_rate_ and *I*_phase_ was 0.640 (*n* = 101 recordings; S3 Fig, panel B, left), indicating that spike phase contributed substantial spatial information (∼56.3%) in excess of firing rate alone. Most of the phaser cell recordings (10/16) with the highest *I*_rate_ values (>0.6 bits/spike) were from hippocampal sites (S3 Fig, panel B, left) and most of those (9/10) were negative phaser cells, consistent with place cells that may have reflected phaser cell activity (Discussion). However, our hippocampal sample was too small to draw clear conclusions. Thus, negative and positive phaser cells may represent diverse spatiotemporal relationships resulting from circuits combining theta-rhythmic inhibition or excitation with varied patterns of spatial drive.

### Statistical models of allocentric factors of spatial activity

Our thesis that phaser cells map spike phase to spatial isocontours (Fig 3D) requires that spiking is predominantly driven by allocentric spatial factors (that is, external cues in a world-centered reference frame). To compare allocentric spatial modulation with other factors, we calculated the spike information content of speed (an idiothetic self-motion signal) and movement direction (an allocentric, but not spatial, signal; Methods). In contrast to the *I*_rate_ comparison, the least-squares optimized slopes between *I*_phase_ and directional (0.086; *n* = 101 recordings) or speed information (0.023; S3 Fig, panel B) indicated minimal coding overlap between *I*_phase_ and other trajectory-based factors. However, it is possible that the spatial modulation apparent in ratemaps (Fig 4) was a spurious by-product of trajectory-based factors and biased spatial sampling of the arena. Firing-rate modulation indices (Methods) for direction (median, 0.379; *n* = 101 recordings) and speed (0.318; S3 Fig, panel C) were suggestive of possible trajectory dependence. Such a confound can result from directionally biased visits to particular locations for which a recorded cell happened to have a similar directional preference. For example, a cell responding to clockwise movement around the arena may produce a spatial ‘wall’ representation if the rat only moved clockwise when contacting the wall.

To isolate spatial-behavioral confounds, we studied a Poisson-distributed generalized linear model (GLM) of spatial (allocentric) and trajectory-based (idiothetic speed, and allocentric non-spatial direction) variables. GLMs have been shown to learn independent spatial and directional contributions to firing that avoid trajectory-driven biases [46, 47]. To capture inhomogeneous changes in spatial or trajectory-dependent selectivity, we fitted GLMs independently to every phaser cell recording for data restricted to sections of a 3 × 3 spatial grid spanning the arena (Methods). The model was trained to predict the spike count for any 300-ms interval *i*

$$\begin{array}{c} {\hat{Y}}_{i}={\hat{\beta }}_{0}+{\hat{\beta }}_{L}L_{i}+{\hat{\beta }}_{Q}Q_{i}+{\hat{\beta }}_{W}W_{i}+{\hat{\beta }}_{S}S_{i}+{\hat{\beta }}_{D}D_{i} \end{array}$$ (1)

where *L* and *Q* are linear and quadratic spatial variables, *W* is a sigmoidal wall-proximity signal, *S* is linear speed, and *D* is movement direction. *L*, *Q*, and *W* are purely spatial whereas *S* and *D* capture the rat’s trajectory as a velocity vector. Thus, we termed this spatial family of GLMs the ‘LQW-SD’ model. To train LQW-SD, we standardized the position and trajectory data from our recordings, but several properties of the data needed to be addressed: (1) statistical dependence among the predictors contributed to an ill-posed problem; (2) spatial predictors had more reliable short-timescale correlations than the trajectory-based predictors; and (3) variable data density across spatial grid segments reduced the validity of model comparisons across the arena. To mitigate these issues, we imposed constraints on model coefficients by training LQW-SD as a ridge regression with *ℓ*_2_- regularization [48]. Further, to maximally expose the spatially inhomogeneous directionality that could have produced behavioral confounds, we chose the regularization penalty that optimized the trade-off between maximizing model directionality and minimizing spike-prediction errors (S4 Fig, panel B+C; Eq (14); Methods). While we did not cross-validate spike-count predictions from the model, our analysis goal was not prediction but to statistically isolate consistent drivers of phaser cell spiking versus spurious factors that may have arisen due to behavioral biases. However, training the model independently within the 3 × 3 grid sections effectively performed a 9-fold cross-validation in space.

We asked whether phaser cell recordings demonstrated directional selectivity that could produce spurious spatial modulation. To quantify directionality, we computed a directional homogeneity index (DHI) on [0, 1] measuring alignment of the 9 *β*_*D*_ vectors (Eq (1)) across the 3 × 3 grid; additionally, we computed a directional strength index (DSI) on [0, 1] measuring the magnitude of *β*_*D*_ relative to the other predictors (Methods). The DHI of phaser cells (median, 0.265; *n* = 69 unique cells with at least one phaser-classified recording) revealed higher homogeneity than nonphaser cells (0.213; *n* = 602; *post hoc* Mann-Whitney *U* = 15, 423, *p* = 0.0005). The DSI of phaser cells (median, 0.0248) and nonphaser cells (0.0127) indicated low overall directionality (*U* = 15, 268, *p* = 0.0003), but it was more widely distributed for nonphaser cells (range, [0, 0.199]) than phaser cells ([0.003, 0.105]). Thus, phaser cells excluded both homogeneous (high DHI, high DSI) and inhomogeneous (low DHI, high DSI) directionality.

**Fig 5:**
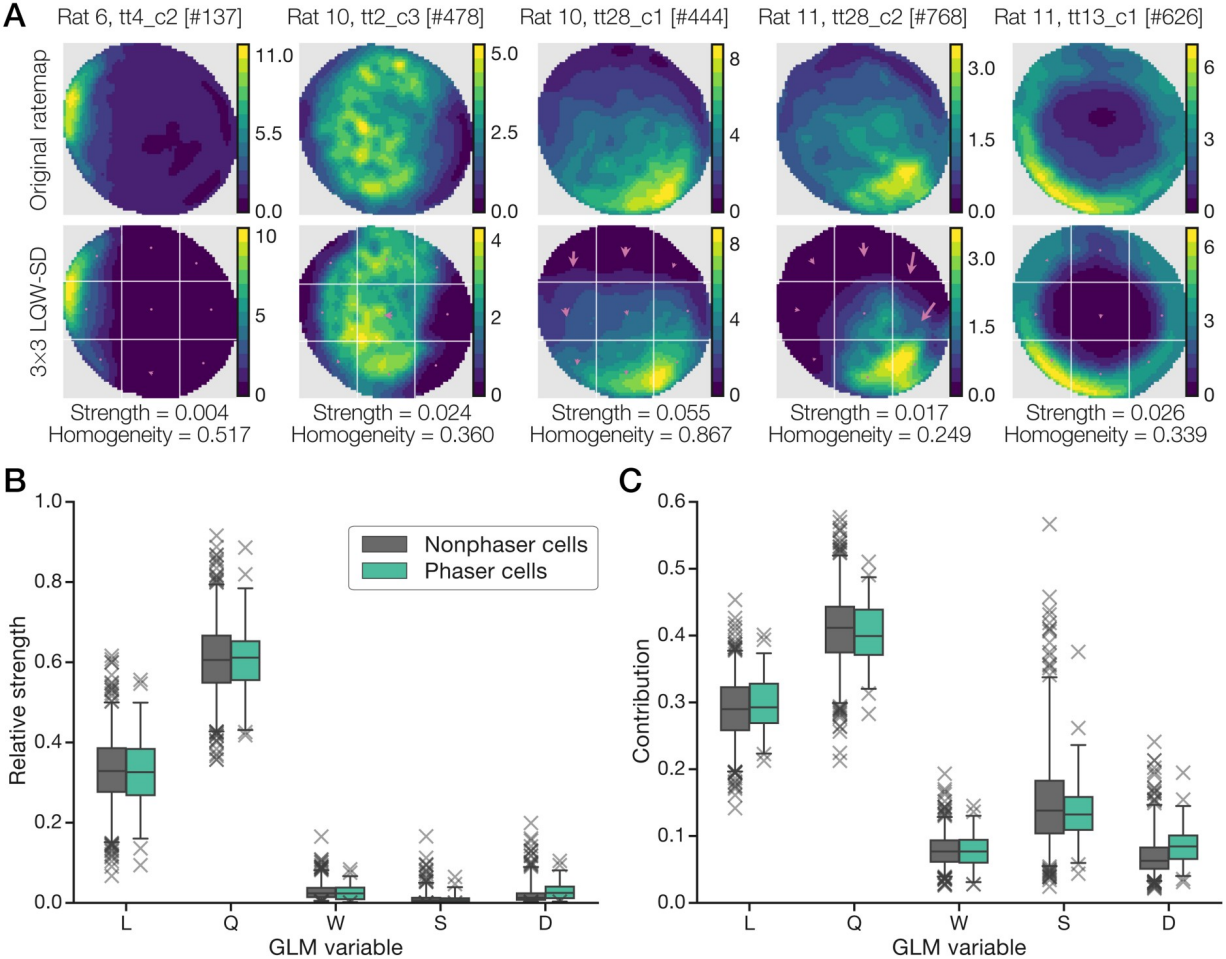
Space–trajectory GLM reproduced allocentric spatial modulation. (A) Actual firing ratemaps (top) and LQW-SD-predicted ratemaps (bottom) for the negative phaser cell examples in Fig 4A. Reconstructions were built from spike-count predictions in each 3 × 3 grid section (Methods). White lines: grid section boundaries; arrows: normalized GLM directional (*D*) weights; Strength: DSI; Homogeneity: DHI. (B+C) GLM spike-count predictions for phaser cells were driven by allocentric spatial variables. The GLM coefficients (B) and maximal contributions (C; Eq (2)) from the spatial (*L*, *Q*, *W*) and trajectory-based (*S*, *D*) variables for phaser and nonphaser cells are shown in 95% box-and-whisker plots with outliers (× markers). For phaser cells, the purely allocentric, second-order spatial predictors (*L* and *Q*) dominated the GLM.

Our analysis was predicated on the ability of the model to explain firing patterns. To verify that LQW-SD could reproduce patterns of spatial modulation, we generated spike-count predictions across the 3 × 3 grid to reconstruct firing ratemaps (Methods). Quantifying accuracy as the vector cosine similarity between ratemaps, we found phaser cells (median, 0.986; *n* = 69 unique cells with at least one phaser-classified recording) and nonphaser cells (0.908; *n* = 602) to have highly accurate reconstructions (*post hoc* Mann-Whitney *U* = 16, 960, *p* = 0.012). Actual and LQW-SD-predicted ratemaps are shown in Fig 5A for the negative phaser cells in Fig 4A with overlaid arrows representing the modeled directionality (*β*_*D*_) of each grid section. To verify that LQW-SD also captured strong directional (high DSI) cells accurately, examples of homogeneous (high DHI) and inhomogeneous (low DHI) directionality are shown in S5 Fig. Thus, LQW-SD provided a high-fidelity account of single-unit firing in our dataset, including spatial and directional cells.

What does the LQW-SD model reveal about spatial vs. trajectory-based predictors? Like DSI for directionality, we computed the relative strength of each model variable (Eq (15); Methods). Box plots (Fig 5B) show the distribution of variable weights for phaser cells (*n* = 69 unique cells with at least one phaser-classified recording) and nonphaser cells (*n* = 602). Both cell types had similar central tendencies with nonphaser cells exhibiting wider ranges of variable strengths. The second-order spatial variables (*L* and *Q*) overwhelmed the wall and trajectory variables, constituting approximately 30% and 60% of the model weight, respectively. Wall/boundary cells were (by inspection) a small number within the dataset, but we considered that the trajectory-based factors (*S* and *D*) might be non-normally distributed, leading to artificially low coefficients. Thus, we computed the importance of model variables by their maximal contribution to predictions over the length of the recording. For variable *X*, we computed its maximal contribution

$$\begin{array}{c} \text{Contribution}\left(X\right)=\max_{i}|\;{\hat{\beta }}_{X}X_{i}\;| \end{array}$$ (2)

across time intervals *i* and sum-normalized the variables (Methods). The contribution profile (Fig 5C) was also dominated by *L* and *Q*, but the *W*, *S*, and *D* contributions were enhanced relative to the strength profile in Fig 5B. Wall and direction variables each constituted ∼8% of the total contribution and nonphaser cells revealed a wide range of speed contributions (Fig 5C, *S*, gray) consistent with the availability of speed signals throughout space-related brain areas [49, 50]. Sorted recording data confirmed this pattern by showing an inverse relationship between spatial and speed-based contributions for phaser cells (S6 Fig); this relationship held for both negative and positive phaser cells (S6 Fig, panel E). Thus, LQW-SD revealed a trade-off between allocentric spatial coding and idiothetic speed modulation, and that phaser cells were overwhelmingly spatial, not directional.

### Approach to modeling LS phaser cells and networks

To gain insight into the possible mechanisms and functions of phaser cell populations, we developed computational models based on minimal dynamics for intrinsic processing of spatial and theta-rhythmic inputs. Crucially, our models assumed that postsynaptic averaging of convergent hippocampal-LS projections produces input to phaser cells that is independent of hippocampus-specific coding (Discussion). Our modeling approach balanced two goals: (1) qualitatively capture salient neurocomputational features of the data, and (2) minimize degrees-of-freedom to avoid model complexity and parameter fine-tuning. Our neuron and network models were broadly tuned to recapitulate several phenomena: (1) theta-bursting rhythmicity (Fig 1A+1D; S2 Fig, panel A, right), (2) symmetric and bidirectional rate-phase coupling (Figs 1E+1F and 3A; S1 Fig, panel E, left), (3) negative/positive phase-shift subtypes (Figs 2A–2C, 3E and 4), (4) temporal segregation of subtypes (Figs 3E+3F and 4), and (5) allocentric phase coding of spatial isocontours (Figs 1B+1C and 2E; S2 Fig, panel B, right; Figs 3D and 5B+5C). Thus, to ensure rhythmicity and realistic spike timing, we based our neuron models on two-variable dynamical systems (Eq (5); Methods) featuring intrinsic bursting dynamics and spike initiation tuned to the activity of hippocampal low-threshold bursters [51, p. 310].

To outline the computational role of phaser cells, our simulations focused on feedforward models in which phasers project to targets that ‘read out’ the phaser cell code. (We will refer to model phaser units as ‘phasers,’ ‘negative phasers,’ or ‘positive phasers’ to distinguish them from our observed ‘phaser cells.’) In the following sections, we present model simulations in several stages: (1) single-neuron phaser models with 1D external inputs, (2) a demonstration model of a small phaser network with artificial 1D spatial inputs and a downstream target cell, and (3) a realistic model of a large phaser network with 2D spatial inputs and several downstream target networks.

### Single-neuron models of negative and positive phasers

**Fig 6:**
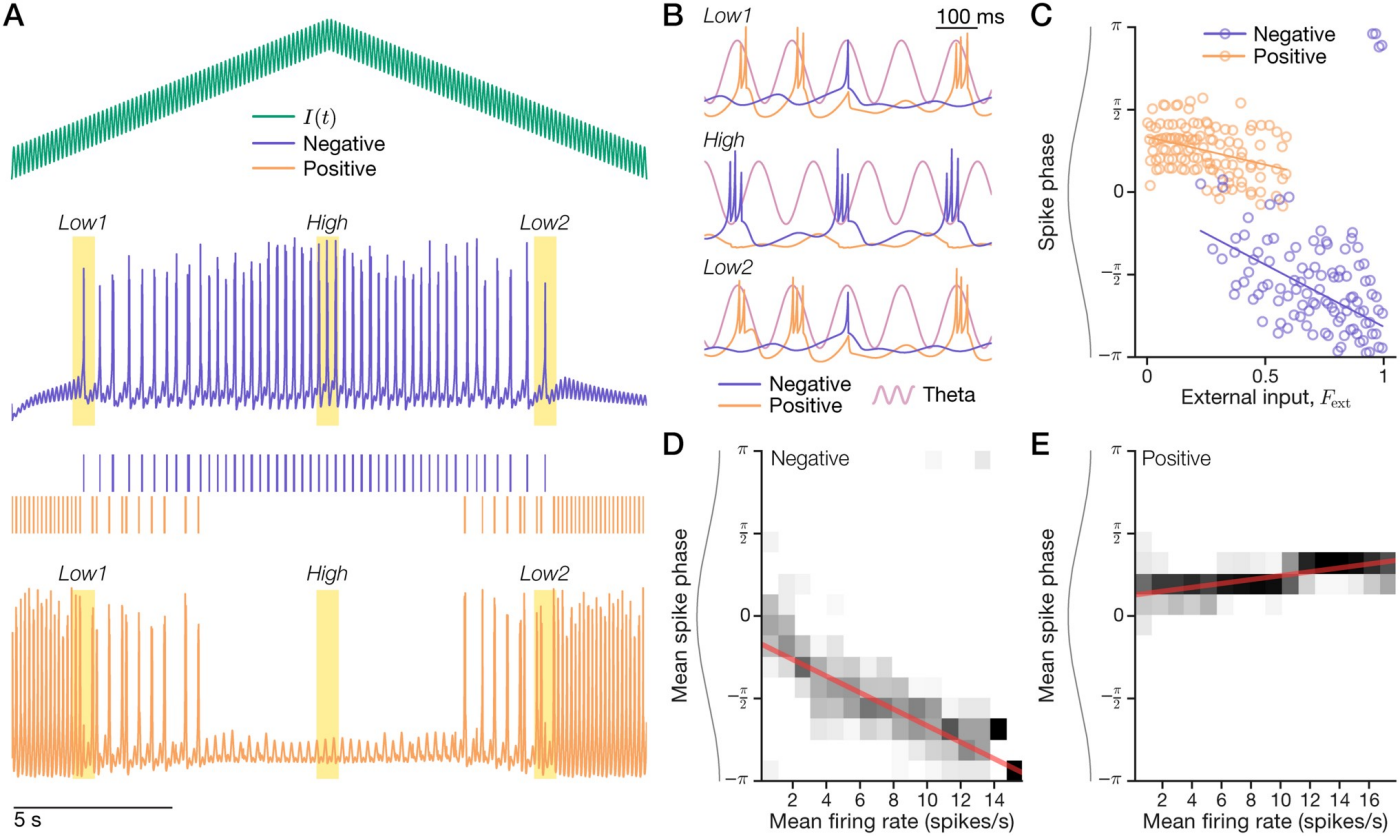
Dynamical models of theta-bursting negative and positive phasers. A model theta-burster (blue, ‘Negative’) with inhibitory theta and excitatory external input (green) provided feedforward inhibition to another theta-burster (orange, ‘Positive’). (A-C) A 20-s simulation. (A) A triangle-wave input (top) produced spiking (*Low1*, *Low2*) and bursting (*High*) in the negative phaser (middle) and a complementary pattern in the positive phaser (bottom). (B) Expanded intervals from the highlights in (A). Sinusoid: the reference theta wave of the simulation. (C) Negative vs. positive phaser spike phase across external input levels. Lines: circular-linear input-phase regressions. (D+E) Rate-phase coupling for the negative (D) and positive (E) phasers. A 1-hr simulation of 10-s to 62-s triangle-wave cycles sampled mean firing rates and mean spike phases for 512 input-level bins. Grayscale: conditional phase distributions; red line: circular-linear rate-phase regressions.

Model negative phasers combined inhibitory theta input and excitatory external input (Eq (6)) with parameters (Tables 2 and 3) that enabled theta-bursting (Methods). Fig 6 shows phaser simulations in which the external input varied up and down over its full range (Eq (8)). For low levels of excitatory input, the negative phaser (Fig 6A+6B, *Low1* and *Low2*) emitted single spikes near theta peak every few theta cycles. For high excitatory input (Fig 6A+6B, *High*), the negative phaser burst with spike triplets near the theta trough on alternating theta cycles. This cycle-skipping rhythmicity is reminiscent of observations in medial entorhinal cortex and the head direction system [52, 53], but this model has no relationship to those phenomena: cycle skipping was a side-effect of the particular theta-bursting parameters (Table 2) that we chose to qualitatively match phaser cell characteristics, which do not include skipping. (The skipped cycles entailed that the resultant spike phase signal was perhaps weaker than if the units had fired every cycle.) Expanded time intervals (Fig 6B) clearly show that the negative phaser shifted to earlier phases of the reference theta wave at high input levels. The model’s rate-phase correlation (*n* = 399/512 nonzero input-level bins; $\hat{r}=-0.809$, $\hat{p}\approx 0$; Fig 6D) revealed strong, consistent phase modulation from peak (0 radians) to trough (−π). That is, spike-phase advanced during rising inputs (Fig 6A; 0–10 s) and then delayed to later timing during falling inputs (10–20 s). The simulated rate-phase coupling is symmetric and bidirectional as predicted (Fig 3C) and it advances to the theta trough as observed for negative phaser cells (Fig 3E).

**Table 2:** Parameters for dynamical theta-bursting neuron models.

Model	*a*	*b*	*c*	*d*	*V*_*t*_	*τ*
Phaser model	0.02	0.2	–50.0	4.0	30.0	7.0 ms
Target burster model	0.02	0.2	–50.0	5.0	30.0	3.0 ms

Parameters (Eq (5); [51]): *a* is an adaptation time-constant; *b* is voltage coupling; *c* is reset voltage; *d* is spike adaptation strength; *V*_*t*_ is the voltage nonlinearity threshold; and *τ* is the membrane time-constant.

**Table 3:** Input and conductance parameters for model phaser units.

Subtype	*g*_*e*_	*g*_*θ*_	*d*_inh_	*E*_inh_	*τ*_inh_
Negative	21.0	–5.0	–	–	–
Positive	–	25.0	3.0	–80.0 (mV)	100 ms

Parameters: *g*_*e*_ is external input gain (Eqs (8) and (4)); *g*_*θ*_ is theta input gain (Eq (7)); *d*_inh_, *E*_inh_, and *τ*_inh_ are the synaptic efficacy, reversal potential, and time constant, respectively, of the negative-to-positive inhibitory conductance (Eqs (9) and (10)).

To model positive phaser cells, we proposed a circuit mechanism whereby a bursting unit driven by excitatory theta input is suppressed by a negative phaser and does not directly receive spatial inputs. We modeled the feedforward inhibition as incrementing a slow 100-ms inhibitory conductance in the positive phaser for each presynaptic spike from the negative phaser (Table 3; Eq (9); Methods). The positive phaser burst at the peak of every theta cycle when disinhibited by low external input to its presynaptic negative phaser (Fig 6A+6B, *Low1* and *Low2*). As the external input rose and fell (Fig 6A), the negative and positive phasers fired in complementary patterns: low/high input silenced the negative/positive phasers (Fig 6C). The model’s rate-phase correlation was indeed positive (*n* = 351/512 nonzero input-level bins; $\hat{r}=0.705$, $\hat{p}\approx 0$; Fig 6E), but weaker and with a shallower phase modulation than both the negative phaser (total phase shift, 0.654 vs. −2.44 radians; Fig 6D) and the positive phaser cell data (∼83% of the low end of the observed range). Positive phaser weakness in the model was commensurate with the higher spatial uncertainty (Fig 2B), lower phase reliability (Fig 2C), and lower phase-code stability (Fig 2G) of positive phaser cells in our dataset. Crucially, negative and positive phasers were temporally segregated according to rate-phase coupling direction (Fig 6D+6E) as in the phaser cell recordings (Fig 3E). Thus, a simple connectivity pattern between theta-bursting models qualitatively recapitulated phaser cell temporal organization.

### Demonstration of a phaser network with artificial 1D inputs

**Fig 7:**
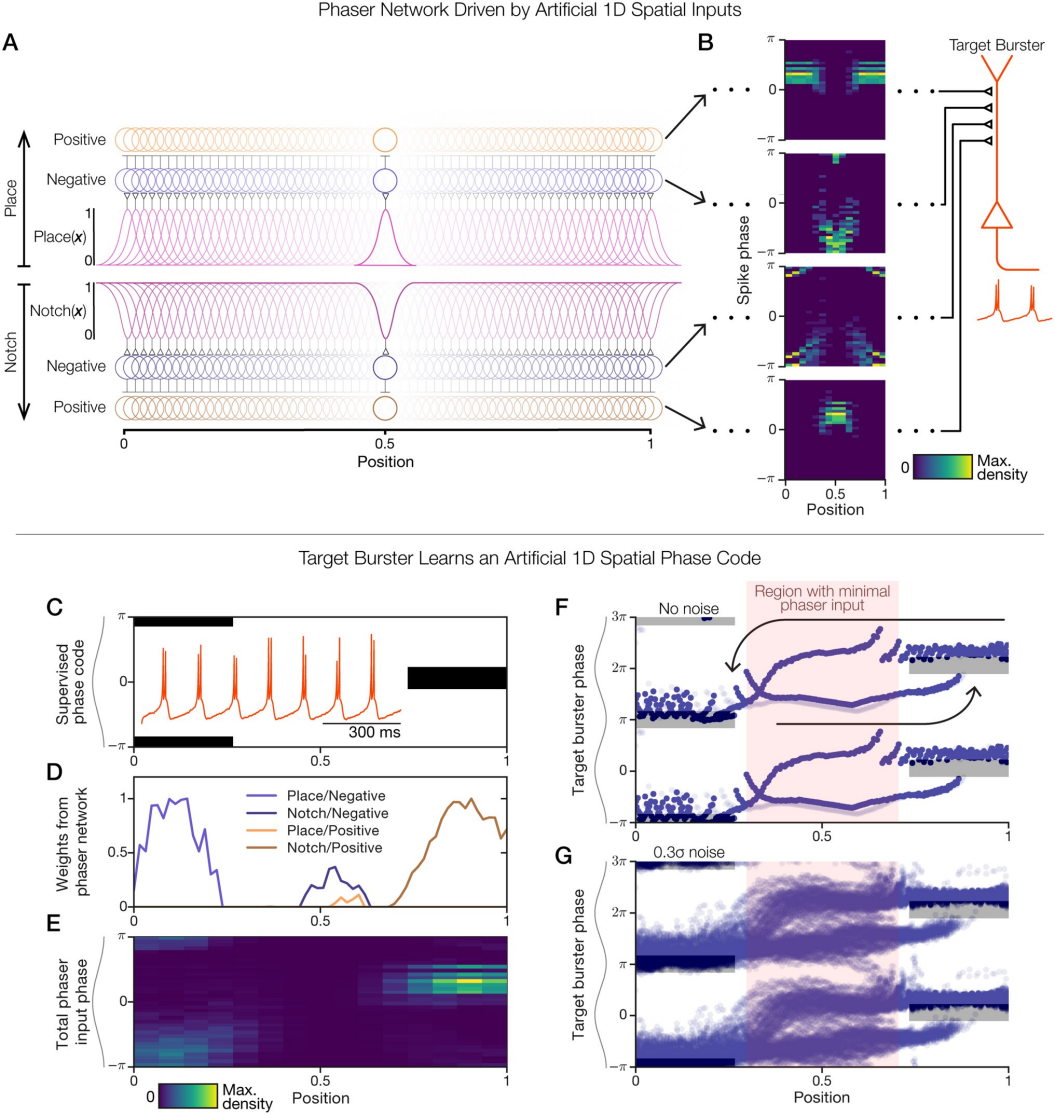
Demonstration of a 1D phaser network and target cell learning. (A+B) We defined a set of 64 place and 64 notch tuning functions as 1D spatial inputs on the range [0, 1] (Methods). (A) Spatial inputs (top, ‘Place’-driven network; bottom, ‘Notch’-driven network) drive 128 pairs of negative (blue circles) and positive (orange circles) phasers. Inputs excite the negative phasers which suppress the positive phasers (Fig 6A). Phasers at position 0.5 are highlighted. (B) A 1-hr simulation sampled spike phase for a 1-min triangle-wave trajectory traversing the space. For the highlighted phasers in (A), joint space-phase distributions of spike timing (left) show the phaser inputs to a downstream target neuron (right). From top to bottom (input/phaser network layer): place/positive, place/negative, notch/negative, and notch/positive. (C-G) Supervised competitive learning over presynaptic phaser inputs trained a ‘target burster’ model (B, right) to follow a spatial phase code. (C) Supervised phase code for training with two modes: theta trough on the left (position 0), theta peak on the right (position 1). Black: desired activity modes; white: untrained. Inset: prior to training, the target burster randomly drifted in phase due to a stochastic input current (Eq (11); S7 Fig, panel D). (D) Competitive *k*WTA weights (Table 4; Methods) for connections from each of the four input/phaser network layers in (A) to the target burster. (E) Total weighted phaser network input to the target burster. (F+G) 1-hr simulations of a 1-min triangle-wave trajectory spanning the range [0, 1]. Target burster output (burst phase) is shown without (F) and with (G) intrinsic noise (*σ*; Table 4). Arrows: phase trajectories for rightward (F, lower arrow) or leftward (F, upper arrow) movement; gray rectangles: supervised phase code from (C); red highlight: region with minimal phaser input based on panels (D) and (E). Multiple theta cycles are shown (*y*-axis) for clarity.

To demonstrate how a downstream target may learn to decode phaser cells, we constructed an artificial 1D spatial paradigm with which to study a model network of 128 negative and 128 positive phasers. The top half of Fig 7 (panels A+B) presents the phaser network and its outputs, and the bottom half of Fig 7 (panels C-G) presents the inputs and outputs of a target neuron model. To emulate the spatial diversity of phaser cells (Fig 4), we created two sets of spatial inputs that each drive one-half of the phaser network: (1) 64 place-like tuning functions (Fig 7A, spatial information flows from the middle to the top of the network diagram), and (2) 64 inverted place-like tuning functions that we termed ‘notch’ functions (Fig 7A, spatial information flows from the middle to the bottom of the network diagram; Methods). A notch function is equivalent to a corresponding place function that has been vertically flipped about its middle so that it is active everywhere except for one location; it is a spatial function and not a frequency filter as the term is used in other domains. Example joint space-phase distributions show the spatiotemporal firing patterns (Fig 7B) that were expressed by the phasers at the spatial mid-point of the network (position 0.5; Fig 7A, highlighted phasers). The four network layers represent the possible combinations of spatial input type (place vs. notch) and phaser subtype (negative vs. positive). These space-phase patterns (Fig 7B), replicated at each of 64 positions across the 1D space, were available as presynaptic inputs to a downstream theta-bursting neuron (‘target burster’; Fig 7B, right). We next demonstrate how this downstream target can utilize phaser activity to learn a spatial phase code.

### Entraining a target cell to an artificial 1D spatial phase code

To demonstrate how phaser inputs can entrain a downstream target, we devised an artificial 1D phase code consisting of two modes: theta-trough timing to the left (position 0) and theta-peak timing to the right (position 1) (Fig 7C). This code associated opposite ends of the 1D space with opposing theta phases. We tuned the target burster model (Table 4; Eq (11); Methods) to emit spike doublets without cycle skipping (Fig 7C, inset; S7 Fig, panel A). Its intrinsic burst rate approximately matched the reference theta frequency (7.5 Hz) of our simulations, but a small deviation caused the burst phase to slowly precess over time (S7 Fig, panel B). That is, the target burster was an intrinsic theta generator independent of other model elements. To amplify its independence, we injected a noisy current (Table 4; Eq (11); S7 Fig, panel C) that caused its burst phase to randomly drift (0.924 angular s.d. over 30 s, *n* = 36 trials; S7 Fig, panel D). To determine feedforward weights from phaser network inputs, we computed the vector cosine similarity between the space-phase distributions of each phaser (as in Fig 7B) and the supervised phase code (Fig 7C). Inputs with the highest similarity were selected by *k*-winners-take-all (*k*WTA; *k* = 25 negative + 25 positive phasers; Table 4; Methods). The resulting weights showed that the theta-trough mode to the left was supported by place/negative phasers, the middle part of the space was not strongly represented, and the theta-peak mode to the right was supported by notch/positive phasers (Fig 7D). The total weighted phaser-network input revealed a qualitative match to the supervised phase code (Fig 7C).

**Table 4:** Input, noise, and learning parameters for target models.

Target model	*I*_const_	*σ*	*g*_neg_	*g*_pos_	*k*WTA
Target burster (1D)	12.65	0.3	1.0	2.0	20% (50/256)
Target burster (2D)	12.65	0.3	10.0	5.0	3.5% (70/2,000)

Parameters: *I*_const_ and *σ* were constant input current and noise gain, respectively (Eq (11)); *g*_neg_ and *g*_pos_ were negative and positive phaser input gains, respectively (Eq (12)).

Note: The *k*WTA column shows the percentage selected, number selected, and total number of competitive synapses.

In a 1-h simulation without injected noise, the target burster’s phase revealed distinct stereotyped phase trajectories for movement to the right or the left (Fig 7F, arrows). Importantly, phaser network activity was not directional (Fig 7B); however, the target burster was directional because its phaser input was effectively released in the middle part of the space (Fig 7D). Thus, in the middle, the target preserved its most recently entrained phase until the simulated spatial trajectory approached the other phase mode. This entrainment dynamic was visibly preserved in a simulation with injected noise (Fig 7G): moving left caused a smooth phase advance to the theta-trough mode, while moving right slowly delayed toward the theta-peak mode until discontinuously jumping ahead of it. The vertical extent of the burst-timing channels at either side (∼*π*/2; Fig 7F+7G) indicated the degree of phase misalignment allowed by this competitive phaser-target burst-synchronization mechanism. While the entrainment did not act perfectly, it prevented the target burster from substantially drifting from the phase code across a range of parameters (S8 Fig). Thus, a phaser network robustly entrained a noisy target cell to a phase code in an artificial 1D space.

### Realistic phaser networks with 2D open-field spatial inputs

To model realistic phaser cell activity, we drove our model phasers (Eqs (5)–(10); Tables 2 and 3; Fig 6) with spatial input functions sampled from a generative model of the open-field spatial modulation of phaser cells (S10 Fig, panel A). The generative sampling model was based on the ‘LQW’ model (Eq (3)), a reduced LQW-SD model that was trained on full recording data (that is, a 1 × 1 grid instead of the 3 × 3 grid) without the trajectory-based variables *S* (speed) or *D* (direction). The result is a seamless model of allocentric spatial selectivity

$$\begin{array}{c} F_{\text{LQW}}\left(x\left(t\right)\right)={\hat{\beta }}_{0}+{\hat{\beta }}_{L}L\left(x\left(t\right)\right)+{\hat{\beta }}_{Q}Q\left(x\left(t\right)\right)+{\hat{\beta }}_{W}W\left(x\left(t\right)\right) \end{array}$$ (3)

for any trajectory *x*(*t*) inside the 80-cm recording arena. In the same way that LQW-SD was optimized to expose directionality (Eq (14); Methods), LQW was optimized to expose wall signals (S4 Fig, panel A) to ensure that the less prevalent boundary/wall responses were captured. The generative model processed and randomized LQW representations to synthesize novel patterns of spatial modulation (S10 Fig, panel A) for negative phasers (as only negative phasers received direct spatial inputs). Given a sampled input function $F_{\text{LQW}}^{*}$, the external input current followed

$$\begin{array}{c} I_{\text{ext}}\left(t\right)=g_{e}F_{\text{LQW}}^{*}\left(x\left(t\right)\right) \end{array}$$ (4)

with excitatory input gain *g*_*e*_ (Eq (8)) and other parameters unchanged (Table 3). We simulated 1,000 pairs of negative (S9 Fig, panel A) and positive (S9 Fig, panel B) phasers, in which the negative phaser inhibited the positive (Eq (9); Fig 6). Simulated phasers expressed place-like, gradient-like, and boundary/wall-like responses (S9 Fig) similar to our phaser cell recordings (Fig 4). We next demonstrate how this realistic phaser network can entrain a downstream target cell.

### Constrained open-field phaser entrainment of single cells

**Fig 8:**
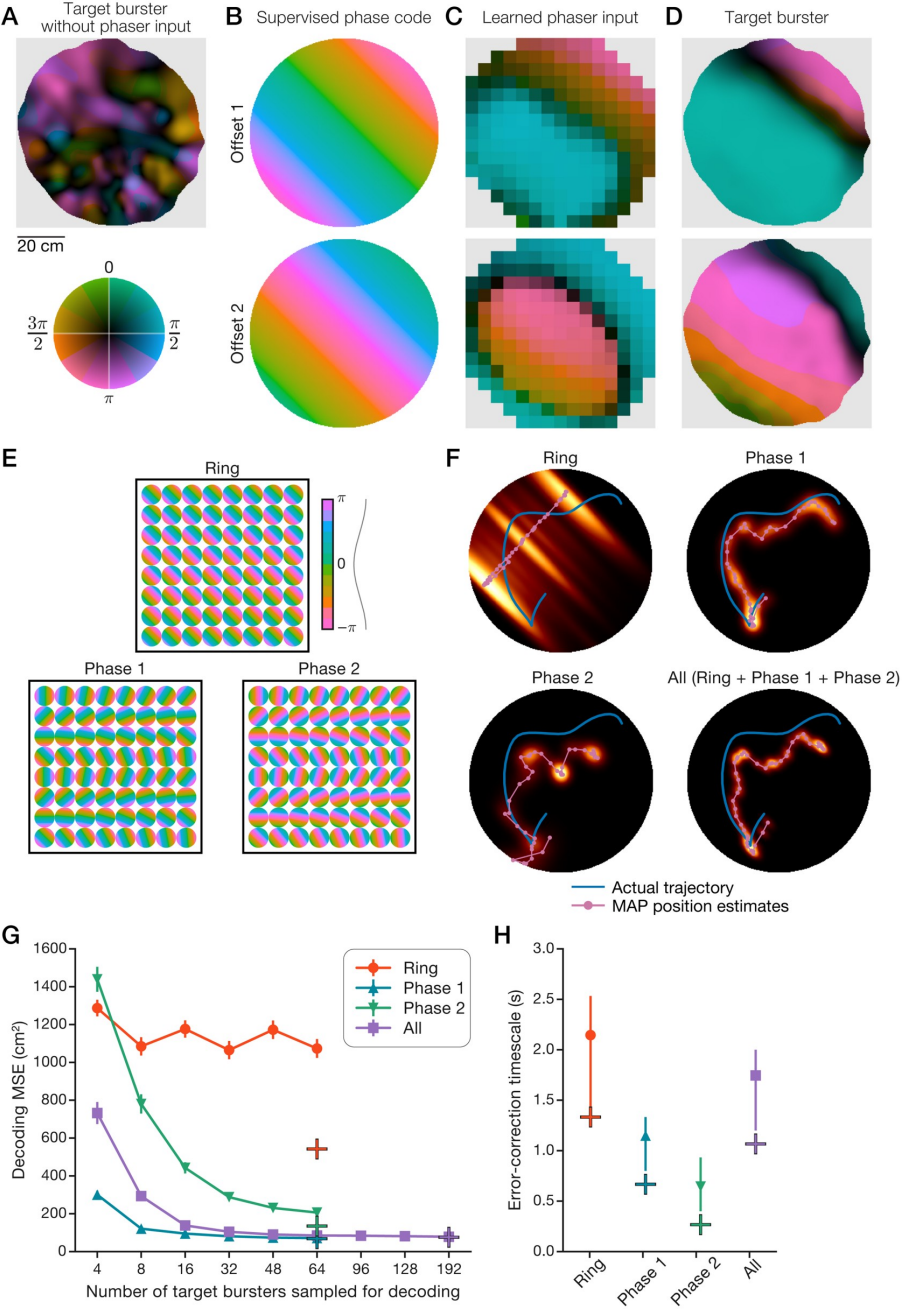
Realistic open-field phaser entrainment of path integration networks. Simulations of 1,000 pairs of negative and positive phasers with generative 2D open-field spatial inputs (S10 Fig, panel A) entrained target cells (A-D) and networks (E-H). (A) Bursting phase map of a target neuron without phaser input. (B) Two supervised 2D phase codes with different phase offsets that emulate oscillatory path integration in the 45° direction. (C) 2D space-phase distributions of total *k*WTA-weighted phaser input to the target neuron (Table 4). (D) Phase maps of the target burster with phaser input. (E-H) Bayesian decoding of position from burst phase (Eq (13); Methods) of three collections of 64 target neurons representing path integration networks. (E) Supervised phase codes for each unit in the target networks. (F) Decoded sequences for an example 6-s trajectory for each target network (64 target units) or the combination of all three networks (192 target units). Heatmap: composited sequential posteriors; magenta line/circles: sequential MAP position estimates; blue line: actual trajectory. (G+H) Path-integration error-correction performance was quantified by decoding a benchmark 60-s trajectory from network activity and 100 bootstrapped unit samples of network activity. Plus symbols: network performance; curves: bootstrap mean; error bars: bootstrap s.e.m. (G) or 95% c.i. (H). (G) Decoded position error according to the number of decoded units. (H) The timescale of error-correction was measured as the HWHM of temporal auto-correlations of decoding error (Methods).

To demonstrate realistic phaser entrainment of a single cell, we simulated a target burster neuron using an actual behavioral trajectory (1 h from Fig 1A). Without phaser input, the target’s bursting phase map illustrated the baseline spatial modulation (Fig 8A; maximum MVL, 0.486) to be expected from a randomly drifting oscillator (S7 Fig, panel D). We devised spatial phase codes representing oscillatory path integration (Discussion) that spanned the arena and the theta cycle. Two such codes with different phase offsets represented path integration of movement in the 45° direction at the scale of the arena (Fig 8B). As in Fig 7D, we calculated the 2D *k*WTA weights (*k* = 35 negative + 35 positive phasers; Table 4) based on spatial phase-tuning similarity between phasers and the supervised phase code. As in Fig 7E, the total weighted phaser-network inputs to the target burster revealed a spatial phase pattern that approximated the desired phase code (Fig 8C). This input pattern comprised a post-theta-peak band (π/2; Fig 8C, top, blue), due to positive phasers, alternating with a theta-trough band (π; Fig 8C, bottom, pink), due to negative phasers; the location of these bands (Fig 8C) tracked corresponding phase stripes in the phase codes (Fig 8B). With phaser input, the target’s phase maps revealed two broad modes of high burst-phase reliability (Fig 8D; bright colors; maximum MVL, 0.994, top; 0.973, bottom) reflecting location-dependent phaser entrainment. The division between the post-theta-peak and theta-trough modes was visibly sharper (Fig 8D, dark stripe) than in the phaser input itself (Fig 8C), suggesting an attractor-like nonlinearity in the input-output phase transformation of phaser-target burst-synchronization. Further, the two entrainment modes were expanded and shifted in the 45° direction relative to phaser input (Fig 8C+8D), analogous to the directionality and delayed onset of entrained bursting observed in the 1D phase trajectories (Fig 7F+7G). Thus, for a single target cell, realistic phasers controlled the spatial distribution of burst timing, but the limited spatial frequency and phase-modulation depth of phaser activity (especially positive phasers, Fig 6E) dynamically constrained the phase-code output.

### Position-coding by collectively entrained target networks

To overcome the constrained output of single target cells, we asked whether a downstream network of multiple cells with phaser inputs would provide a stronger position signal. We considered target networks to be simple collections of target burster units (Eq (11); Table 4); each unit had its own set of competitive synapses carrying input from the 2D phaser network. We constructed three target collections of 64 units (Fig 8E; S10 Fig, panel B). By analogy with oscillatory ring-attractor models of path integration [26, 27], we created the ‘Ring’ collection with identical preferred directions but a full range [0, 2π] of phase offsets (Fig 8E, top). Because a single ring network is directionally biased, we expected that it would not support a clear open-field position signal on its own. The remaining two collections were constructed with a full range [0, 2π] of preferred directions but identical phase offsets across units (Fig 8E, bottom). These collections, ‘Phase 1’ and ‘Phase 2,’ were equivalent to taking a single-phase slice across a population of ring attractor networks (S10 Fig, panel B). For each collection, every unit’s phase code (Fig 8E) was learned via *k*WTA competition and simulated with a 600-s behavioral trajectory. Due to the feedforward phaser-target connectivity, all units were simultaneously entrained by the same open-field phaser network (as in Fig 8C+8D). The phaser input and unit output maps are shown as movies for the Ring (S1 Movie), Phase 1 (S2 Movie), and Phase 2 (S3 Movie) collections. Thus, realistic 2D phasers enabled functionally flexible phase-code entrainment of many downstream targets.

To uncover the collective position signal in these collections, we applied the method of Bayesian spike-count decoding of position [54] to the phase domain (Eq (13)) to infer estimated trajectories from simulated burst timing (Methods). If this position signal were to support the resetting of path integration, then it should be quantified in terms of position-error correction. Example 6-s trajectories with maximum a posteriori (MAP) estimates of position revealed that, as expected, the Ring network poorly tracked the trajectory (Fig 8F, top left), but the Phase 1 and Phase 2 collections more closely approximated the trajectory’s position and shape (Fig 8F, top right and bottom). To quantify error correction, we decoded a benchmark trajectory across collections and bootstrap unit samples (Methods). The mean squared error (MSE), based on the distance between actual positions and MAP estimates (Methods), showed that the Ring network consistently performed poorly, but the Phase 1 and Phase 2 collections’ performance substantially improved by collectively decoding larger numbers of units up to the total of 64 (Fig 8G). Phase 1, Phase 2, and the combination of all collections exhibited average decoding errors of 8.25, 11.6, and 8.70 cm, respectively.

### Timescale of path integration error-correction

To be useful, phase resets should occur quickly. To measure the timescale of error-correction in phaser-entrained targets, we computed temporal auto-correlations of decoding errors for the benchmark trajectory (S10 Fig, panel C). We quantified the typical timescale of error-correction as the correlation’s half-width at half-maximum (HWHM; Methods). Across target collections, the HWHM timescale (Fig 8H) revealed subsecond correction in the Phase 1 (0.667 s) and Phase 2 (0.267 s) collections and 1-second correction in the combined collection (1.067 s). In our framework, correcting path integration errors depended on populations of ring networks (as represented by the Phase collections) or other structures with diverse preferred directions. As expected, a single ring network (or other directionally homogeneous integrator) would be insufficient to support a 2D position signal. Further, our target units were not performing path integration: they were noisy, intrinsic theta-bursters. Thus, error-correction performance in our models provided a lower bound: presumably, a path-integrating target would have fewer errors to correct than randomly drifting oscillators.

## Discussion

We recorded single-units from freely exploring rats in septal, hippocampal, thalamic, midbrain, and other brain areas and found neurons in LS and the hippocampus whose spiking theta-phase was symmetrically and bidirectionally coupled to spatial modulations of firing rate. Tight rate-phase coupling entailed that spike phase mapped to isocontour levels of spatial inputs. We theorized that phaser cells serve to transform spatial information into the temporal-phase domain for downstream spatial computations. Phaser cells exhibited negative (phase advance) or positive (phase delay) modulation for increasing firing rates. Temporal segregation of negative and positive phaser cell activity was consistent with experience-independent phase-coding mechanisms and our models’ assumptions of inhibitory/excitatory theta input to negative/positive phaser cells. We trained space–trajectory GLMs to verify that phaser cell spiking was overwhelmingly driven by allocentric spatial factors and not spatially inhomogeneous modulation by speed or movement direction. We asked what mechanisms could explain the spatiotemporal organization of phaser cells and what functions they could serve in LS output targets. We demonstrated minimal circuit models of bursting neurons that qualitatively accounted for our main observations. In artificial 1D and realistic 2D open-field spatial simulations, we showed that phaser networks collectively entrained target neurons and networks to spatial phase codes using a competitive learning rule. Moreover, Bayesian position decoding of simulated burst phase in phaser-entrained targets revealed a strong, error-correcting spatial signal organized by location-dependent synchrony. Our results suggest a framework in which LS spatial phase representations enable flexible computations of spatial synchrony in subcortical networks interconnected with the hippocampal formation.

### Spatial phase codes in the hippocampus and lateral septum

Hippocampal place fields [55] were studied extensively as a spatial firing-rate code prior to the characterization of spike theta-phase precession [4, 6, 56]. Theoretical models and in vivo manipulations have explored how interacting oscillations, ramp currents, or intrinsic dynamics may account for the link between phase precession and firing rate [7–12]. An analysis of pooled hippocampal activity highlighted the asymmetry of phase precession (Fig 3B) by finding clear theta coupling before the animal entered the classical rate-based place field [43]. This extended oscillatory coupling may reflect a critical role for phase precession in compressing place cell activity [57] into the timescale of synaptic plasticity [58, 59]. If phase precession is primarily involved in the internal temporal organization of place cell activity, then spatial and theta-rhythmic input from the hippocampus may be transformed for other functions by other brain areas.

Our analysis characterized the rate-coupled phase code of phaser cells as distinct from hippocampal phase precession. Most phaser cells in our dataset were located in LS (Table 1), a primary subcortical target of dense, convergent hippocampal efferents [42, 60] that had previously been shown to carry a degraded spatial rate code [39–41]. Tingley & Buzsáki (2018) [42] reported that many LS neurons recorded during track running carried spatial phase codes that were similar to phase precession except for rate independence and larger spatial extents than typical place fields. Their analysis [42] indicated that the LS phase code depended specifically on hippocampal phase precession coordinating theta sequences in CA3 and CA1 inputs. However, this leaves open the questions of what LS phase codes in the open field look like and whether previously described LS rate-coding neurons also carry a phase code. Examining a single open-field behavioral condition, we found that 15.6% (50/321) of LS neurons yielded phaser-classified recordings according to our criteria (16 medial septal cells were not phaser cells; Table 1). Unlike the Tingley & Buzsáki [42] phase code on tracks, LS phaser cells had strongly rate-coupled phase modulation and a wide range of spatial patterns including wall/boundary responses [61–63] that may be available to the LS via subicular afferents [60]. LS phaser cells demonstrated a symmetric and bidirectional code for allocentric space (Fig 3A), whereas hippocampal phase precession is an asymmetric and unidirectional code for distance relative to the boundaries of a place field (Fig 3B). Thus, rate-coupled phaser cells and rate-independent precession may represent distinct neuronal populations or distinct operating modes within LS and/or other structures, possibly mediated by heterogeneous connectivity patterns.

Delay-based phase codes as in our positive phaser cells have not, to our knowledge, been previously demonstrated. Three of our positive phaser cells were located in the dentate gyrus, which receives input from a LS-supramammillary pathway [60], suggesting possible hippocampal entrainment by LS phaser cell activity. Hippocampal negative phaser cells with strong spatial rate codes (and place-like selectivity) additionally demonstrated stronger directional and speed coding (S3 Fig, panel B), thus contributing to the trajectory component of the space–trajectory trade-off observed in our GLM analysis (S6 Fig). Our sample of hippocampal cells was too small to draw conclusions, but that relationship suggests that some hippocampal phaser cells may have been place cells reflecting phaser-entrainment signals from subcortical pathways. Our positive phaser model was based on theta excitation and negative-phaser inhibition (Fig 7), consistent with the prevalence of GABAergic neurons and recurrent collaterals in LS [60]. Our bursting models showed that, given convergent spatial and theta-rhythmic input, phaser cells could operate intrinsically without inheriting phase relationships from CA3 or CA1. Convergent inputs allow the possibility that the longitudinal-to-vertical-band topography of the hippocampus-LS projection [60] averages over the spatial and theta-rhythmic activity of many place cells, effectively displacing hippocampal tuning specificity so phaser cells can exploit hippocampal input while computing distinct codes. Thus, both extrinsic and intrinsic phase transformations of hippocampal spatial information may arise in the LS and/or other structures depending on contextual and behavioral requirements.

### Theta oscillations for the future and the present

Early theoretical models suggested that hippocampal sequences, learned via phase precession and/or temporally asymmetric synaptic plasticity, enabled context-dependent predictions of future positions [64–68]. Experimental studies revealed theta-rhythmic forward-sweeping sequences during active locomotion [69, 70] that mentally probed paths ahead of the animal’s current position to guide navigational decisions [71, 72]. This research suggests a major function of theta-rhythmic information processing along the trisynaptic circuit of the hippocampal formation is to generate memory-guided predictions of future states given the current state. The current state may be reflected in CA3 or CA1 activity at the trough of local theta waves [56], but it could also be directly encoded by other theta-rhythmic structures. Specifically, if recurrent network plasticity and phase precession enable future-oriented sequences, then phase codes in extrahippocampal circuits without those elements may be more likely to encode the current state by default. Such phase codes would be symmetric and bidirectional, similar to phaser cells as well as hippocampal place fields during initial exposure to a novel environment [8, 44, 73]. Thus, phaser cells may provide an experience-independent temporal code for the current state.

The phaser cell spatial transformation is inherently less precise than phase precession. Its bidirectionality assigns the same phase to different locations: for example, a single phase would map to opposite edges of a 1D place field on a track (Fig 3C) or a concentric ring (isocontour) of a 2D place field (Fig 3D). In contrast, the unidirectionality of phase precession enhances the rate-coded position signal of a place cell by contributing unambiguous information about distance traveled through its place field [4, 6]. Phase precession constructively adds to coding precision, but the phaser cell code may serve to directly transform spatial information. We showed that the phaser cell code was stable across hours and days, suggesting that it may contribute to the context-dependent spatial computations of hippocampal/entorhinal circuits. LS spatial modulation has been previously shown to exhibit distinct responses to context changes compared to hippocampal place cell remapping [41]. Our study did not address context-dependence, but it did reveal spatial heterogeneity across phaser cells (for example, Fig 4), thus supporting our theoretical notion that phaser cell responses provide a basis for flexible spatial learning across contexts.

One benefit of a bidirectional phase code is that positive phase modulation can coexist with negative phase modulation in the same network. To illustrate the spatiotemporal activation of symmetric rate-coupled phase codes, we could imagine layers of negative and positive phaser cells with 2D bell-shaped spatial tuning and uniformly distributed fields. At the trough of a theta wave, negative phaser cells representing the current location fire first and strongest, followed by their neighbors in all directions. Activation continues in a radial wave extending outward and dissipating by theta peak. Positive phaser cells, conversely, follow a reverse radial wave that begins with a wide concentric circle of weak firing at theta peak and collapses onto the current location with strong firing before theta trough. This expansion and contraction of radial waves would collectively span the theta cycle as a consequence of the theta-segregation of negative and positive phaser cells (Fig 3E). Thus, phaser cells may form a spatiotemporal cursor marking the present.

### Path integration reset by subcortical entrainment

The neural mechanisms of path integration are not well understood. In rats, experimental inactivation of the medial septum has been shown to reduce the theta rhythm and disrupt grid cell firing [74, 75], but preserve the spatial firing of hippocampal place cells [76] except in conditions such as large environments (or wheel running) in which performance would be expected to rely more on path (or time) integration than external cues [77]. Similarly, septal inactivation of theta using gabazine (but not muscimol or tetracaine) was demonstrated to preserve hippocampal spatial activity while impairing navigation to a hidden goal [78]. In mice, path integrating behavior is preserved in the dark (cf. the control animals tested by [36]) even though spatial grid cell activity has been shown to require visual input [35]. These findings suggest the theta rhythm is critical to path integration independent of place field maps or grid cell periodicity, raising the question whether it plays a direct computational role or a supporting role (such as phase reset), or contributes to both.

The temporal interference models based on VCO units [19–21] posited a direct role in which relative phases between oscillators constitute a spatial vector anchored to a previous reference point. We previously showed that a generalized VCO model could be effectively calibrated by extended sensory cue interactions that mediated phase-code feedback [31], although that study was agnostic to the feedback mechanism. Here, we demonstrated burst-synchronized entrainment of target neurons that learned VCO-like activity patterns (Fig 8). However, detecting collective synchrony among a population of phaser cells is a general decoding mechanism that could theoretically support a continuous attractor network of grid cell activity [30]. In that case, temporal coordination within the theta cycle might act as a signal boost for spatial feedback to reset the location of the activity bump (cf. [32]). Additionally, a main criticism of VCO theories followed from the finding of grid cells in bats without continuous theta oscillations [79]. However, like VCO-based path integration (see discussion in [25]), a phaser-based reset does not necessarily require rhythmic periodicity: synchrony could arise from structured latencies due to shared arrythmic inputs. Indeed, phase locking and phase coding by hippocampal and medial entorhinal neurons in crawling bats has been reported to be organized by nonoscillatory LFP fluctuations [80]. While our phaser models required theta rhythmicity, the mechanism of spatial synchrony that they demonstrated could be generalized to nonoscillatory systems. Despite widely varying navigational and perceptual requirements across species, synchronous (but not necessarily oscillatory) neural activation may be organized by allocentric features. The main requirement is that path integration reset must be linked to the current state of the world. Thus, LS phaser cells in rats may operate a present-focused reset mechanism parallel to future-focused hippocampal dynamics.

### Learning to reset internal states with external cues

The phaser models assumed that temporal contiguity, as measured by spatial phase-tuning similarity, promotes associative synaptic weights [58, 59] between phaser cells and their targets. The supervised competitive mechanism was not realistic, but our modeling goal was to demonstrate the functional implications of having competitively weighted phaser inputs. The simplified learning mechanism represented the end result of an animal’s familiarization with a given environment. During exploration, we supposed that path integration produces a ‘teacher’ signal that associates internal states with external cues represented in phaser cell inputs. This would be a noisy signal in novel environments or disoriented animals, but investigatory behaviors in those situations emphasize incremental exploration and active management of path integration [81]: shorter excursions, direct returns to home base, and more visual fixations and/or head scanning [82]. These behaviors may stabilize the teacher signal to allow the path integrator to learn new weights from phaser cells (or other inputs). For example, in a VCO-based path integrator, relative phases between ring networks would coherently advance and delay relative to idiothetic motion signals [26, 27]. As long as those phase modulations were relatively continuous between sensory fixations, then any resulting spatial structure in the relative phase pattern would serve to reinforce itself by enhancing co-active inputs from phaser cells with similar spatial phase tuning. Our supervised phase codes (Figs 7C and 8B+8E) temporally collapsed the process of learning a teacher signal into a single pattern.

An additional complication for VCO-based path integration is that learning requires theta-rhythmic coupling between the target and its phaser inputs. However, the burst frequency of VCOs increases with movement in the preferred direction [19, 22]. Thus, phase-coupled synaptic modification would be restricted to the subset of VCOs with preferred directions orthogonal to the animal’s current direction. This limitation would be mitigated by ring attractor organization of VCO cells [26, 27], in which learning would be continuous because every orthogonal direction would be represented by a cell in the network. For continuous attractor-based path integration in grid cells, phaser cells and grid cells would be phase coupled via the shared hippocampal-entorhinal theta rhythm [83], but phase locking of layer III grid cells to the local theta trough [5] could restrict learning to negative phaser cell inputs. Future studies are needed to determine biologically plausible learning mechanisms.

The continuous activity of phaser cells further raises the question of how a path integrator would switch from internally integrating self-motion to receiving phase-code feedback to reset errors. Presumably, both processes could not occur concurrently. Our models (including [31]) suggest that resetting to stabilize the spatial representation of a familiar environment requires theta-phase coupling (similarly to learning) but it only needs to punctuate path integration briefly enough to achieve burst synchronization (Fig 8H; S7 Fig). Punctuated resets could be adaptively driven by investigatory behaviors like head scanning [82] or boundary visits [84], or by error signals mediated by grid cells [27, 85]. Ring attractor organization of VCOs could enhance the robustness of phase-code resets by propagating updated phase offsets via intrinsic connectivity. Furthermore, our examination of LS phase codes may be biased by our sample of recording sites. Tingley & Buzsáki (2018) [42] found a dorsal-ventral dissociation in LS phase coding properties, including evidence that local theta is a traveling wave in the dorsal-ventral and medial-lateral directions. Thus, the theta-phase diversity of phaser cells is potentially much broader than our sample, enabling additional entrainment or switching mechanisms in downstream targets.

### Concluding remarks

Theories of the neural circuits of spatial cognition should go beyond representations to describe how target brain areas read, decode, and translate signals along the path to decisions and behavior. We presented exploratory single-unit data revealing a rate-coupled spatial phase code in neurons found in the LS, hippocampus, and other subcortical areas. Dynamical bursting models helped to explain observations in the data, but they also demonstrated how collective synchronization codes among phaser cells could be learned and decoded by target cells and networks. Our data and models suggest a subcortical phase-code feedback loop for allocentric space may be mediated by phaser cells in LS and/or other regions. Future studies of the role of theta oscillations in spatial navigation may consider the phaser cell mechanism or our theorized feedback pathway to provide a useful perspective. Further research is needed to determine which pathways might support this feedback, but the LS is ideally positioned to translate hippocampal spatial and theta-rhythmic output to downstream subcortical areas [60, 86] that regulate the theta rhythm [37, 38] and theta-bursting thalamic nuclei [22, 87, 88] including the nucleus reuniens with hippocampal and entorhinal projections [60, 89, 90]. Spatial synchronization codes may resonate through limbic loops to reconcile internal maps with external sensory experience.

## Methods

### Ethics statement

Rats were chronically implanted with recording devices under deep isoflurane anesthesia. All experiments were conducted in accordance with the U.S. National Institute of Health Guide for the Care and Use of Laboratory Animals (NIH Publications No. 90-23), and were approved in advance by the animal subjects review committee at the University of California, Los Angeles.

### Bursting models

We define a quadratic integrate-and-fire model [51] of intrinsic bursting with a fast variable for the spiking limit cycle (*V*) and a slow adaptive variable for terminating bursts (*u*). The dynamics follow

$$\begin{array}{c} \tau \dot{V}=\Phi (V)-u+I(t)\\ \tau \dot{u}=a(bV-u) \end{array}$$ (5)

where *I*(*t*) is a cell-specific time-varying input, Φ(*V*) = 0.04*V*^2^ + 5*V* + 140 is a quadratic nonlinearity for spike initiation, *a* and *b* control adaptive feedback, and *τ* sets a shared time-scale for spiking and bursting (in addition to the time constants implicit in Φ(*V*) and *a*). Whenever *V* > *V*_*t*_, a spike is recorded, *V* is reset to *c*, and *u* is incremented by *d*. Bursting parameters are listed in Table 2. While *V* is approximately millivolt scale, we treat this system as a qualitative, not biophysical, model for which the parameters are in arbitrary units. For theta-rhythmic inputs and recording theta phase, simulations tracked a reference theta wave at frequency *f*_*θ*_ = 7.5 Hz, matching the typical burst rate in our single-unit recordings.

For negative phasers, we set the time-varying input (Eq (5)) to the combination

$$\begin{array}{c} I\left(t\right)=I_{\theta }\left(t\right)+I_{\text{ext}}\left(t\right) \end{array}$$ (6)

of sinusoidal theta inhibition (for inhibitory gain *g*_*θ*_ < 0)

$$\begin{array}{c} I_{\theta }\left(t\right)=g_{\theta }\;\left[0.5\;\left(\cos\left(2\pi f_{\theta }t\right)+1\right)\right] \end{array}$$ (7)

and external excitatory input (for excitatory gain *g*_*e*_)

$$\begin{array}{c} I_{\text{ext}}\left(t\right)=g_{e}F_{\text{ext}}\left(t\right) \end{array}$$ (8)

where the external input function *F*_ext_(*t*) had range [0, 1].

The positive phasers had theta gain *g*_*θ*_ > 0 and followed Eq (5) with negative-phaser input

$$\begin{array}{c} I\left(t\right)=I_{\text{neg}}=-g_{\text{inh}}(V-E_{\text{inh}}) \end{array}$$ (9)

where *g*_inh_ was a slow inhibitory conductance

$$\begin{array}{c} \tau_{\text{inh}}\;{\dot{g}}_{\text{inh}}=-g_{\text{inh}} \end{array}$$ (10)

that was incremented by *d*_inh_ with every pre-synaptic spike (Table 3).

The target bursters had a shorter time-constant (↓*τ*) and lower burst excitability (↑*d*; Table 2). In place of Eq (5), the fast variable followed

$$\begin{array}{c} \tau \frac{dV}{dt}=\Phi \left(V\right)-u+I_{\text{syn}}\left(t\right)+I_{\text{const}}+\sigma \xi \frac{\tau }{\sqrt{dt}} \end{array}$$ (11)

where normalized white noise *ξ* was controlled by gain *σ*, and *I*_syn_(*t*) was the total synaptic drive from the phaser network

$$\begin{array}{c} I_{\text{syn}}\left(t\right)=\sum_{k\in \{\text{neg},\text{pos}\}}[g_{k}\sum_{j=1}^{n_{p}}W_{k}^{j}\delta \left(t-t_{k}^{j}\right)] \end{array}$$ (12)

where *n*_*p*_ was the number of phasers in each subtype layer, *g*_neg_ and *g*_pos_ were subtype-specific feedback gains (Table 4), *W*_neg_ and *W*_pos_ were the phaser weight vectors (for example, Fig 7B), and *t*_neg_ and *t*_pos_ were most-recent-spike vectors. Constant input current was tuned (*I*_const_, Table 4) so that the intrinsic burst rate, without noise or synaptic input, was close to reference theta frequency (7.519 s^−1^ compared to *f*_*θ*_ = 7.5 Hz).

### Spiking simulations

Spiking neuron and network models were implemented in the equation-based Brian simulator [91]. Simulations were integrated in 1-ms timesteps. Phaser layers and the target burster without noise were evolved with Runge-Kutta 4th-order integration; the target burster with noise used the Euler-Maruyama method. Burst timing in simulations was determined as spike times following interspike intervals ≥ 25 ms.

For 1D spatial simulations, place tuning functions were Gaussian functions with bandwidth 1/64 normalized to the range [0, 1] and centered at 64 evenly-spaced positions from 0 to 1. Each notch tuning function was 1 minus a place tuning function. The gain of phaser input onto the target burster (Table 4) was manually tuned for visually matched ‘middle of the road’ synchronization at both fixed points.

For 2D spatial simulations, phase code gratings had 80-cm spatial periods so that one cycle covered the environment. Phaser gain onto the target burster (Table 4) was manually tuned to roughly equalize the size of negative and positive synchronization modes across different reference phases.

### Competitive learning

Based on 1-hr training simulations, we generated joint space-phase distributions from phaser spikes: 15 × 36 (*x* × *ϕ*) bins for 1D simulations; 15 × 15 × 36 (*x* × *y* × *ϕ*) bins for 2D simulations. The supervised phase code was either directly specified as a binary array for 1D simulations or binned from a spatial grating function for 2D simulations. We computed the vector cosine similarity between the space-phase distributions of the phasers and the supervised phase code as the basis for feedforward synaptic weights from the phaser layers to the target burster. To determine competitive weights, we chose the *k*WTA negative and *k*WTA positive phasers (Table 4) with the highest similarities and normalized those similarities to the range [0, 1] via [(similarity − min)/(max − min)]. Inactive weights were set to 0. Total phaser input (Figs 7E and 8C) was computed as the product-sum of the weight vector and an array of all space-phase distributions.

### Bayesian phase decoding

We simulated target networks with 64 bursting units that each learned different ranges of phase offsets and preferred directions (Fig 8E). Burst timing was decoded in 267-ms sliding windows (2 theta cycles) that were incremented in 133-ms steps (1 theta cycle). For each unit, the average burst phase was computed in each window; the previous average was used if no bursts occurred in the window. Analogous to methods for decoding spike counts [54], we calculated the posterior probability distribution of spatial position *P*(*x*|*ϕ*) for an array of phase values *ϕ* as

$$\begin{array}{c} P\left(x|\phi \right)=P\left(x_{t}|\phi ,{\hat{x}}_{t-1}\right)=C\left(\tau ,\phi \right)\exp(\frac{-\left|\right|{\hat{x}}_{t-1}-x_{t}\left|\right|}{2\sigma_{c}^{2}})\prod_{i=1}^{n}\exp\left(\cos\left(\phi_{i}-\Phi_{x,i}\right)\right) \end{array}$$ (13)

where *x*_*t*_ was the position for the current window, ${\hat{x}}_{t-1}$ was the MAP position estimate for the previous window, C was a normalization factor based on *ϕ* and window-size *τ* that ensured ∑_*x*_ *P*(*x*|*ϕ*) = 1, *σ*_*c*_ = 15 cm was the Gaussian width of a spatial contiguity prior, *n* was the number of units, and Φ_*x*,*i*_ was the phase value at position *x* of the 2D spatial phase code that was used to train unit *i*.

Decoding MSE was computed as the mean squared Euclidean distance between the MAP position and the average of recorded trajectory samples within each window across a 60-s trajectory segment used as a performance benchmark. We decoded the activity from three target-burster networks with 64 units (Fig 8E; S10 Fig, panel B) and the combination of all three networks with 192 units. Each network condition was bootstrapped by sampling (or subsampling to smaller network sizes as in Fig 8G) with replacement the units in the network and then decoding the sample’s activity and computing the MSE as described. Temporal autocorrelations (S10 Fig, panel C) were computed using full-size networks (64 or 192 units) by correlating each bootstrap MSE time-series with itself and normalizing the minimum and maximum of the mean bootstrap correlations to [0, 1]. HWHMs were calculated as the time lag of the earliest window with normalized correlation <0.5 for each bootstrap; data are shown (Fig 8H) as means and empirical 95% confidence intervals of bootstrap HWHMs.

### Subjects and surgery

Male Long-Evans rats (350–400 g) were individually housed and kept at 85% of ad libitum weight. They were trained over 5 d to forage for food pellets in an enclosed environment. Under deep isoflurane anesthesia, rats were chronically implanted with tetrode arrays targeting (across rats) the medial and lateral septum, dorsal hippocampus, anterior thalamus, midbrain, and/or other subcortical areas. Each rat was implanted with 16 tetrodes (64 electrode channels) that were grouped into four independently drivable bundles of four tetrodes each.

### Single-unit recordings

Data collection methods including conduct of recording sessions, video tracking analysis, and single-unit acquisition have been described previously [22]. Spike trains recorded during different sessions were considered to be from the same cell if (1) they were obtained from the same tetrode, (2) the tetrode had been advanced <80 *μ*m between recordings, and (3) cluster boundaries and waveform shapes were visually similar on all tetrode channels for both sessions. The phase of the septal-hippocampal theta oscillation was quantified from the LFP signal on a reference electrode in the hippocampal stratum oriens. In one subject (rat 11), a strong theta-rhythmic cell was used as phase reference instead of the LFP signal and was not included in data analysis. All analysis data was filtered for linear movement speeds >5 cm/s.

### Adaptive Gaussian-kernel spatial maps

To handle large variance in spatial data density from long recordings, we computed spatial maps with adaptive scaling kernels. We used a KD-tree algorithm to generate a nearest-neighbor model of the data points for the map. For every pixel to evaluate, we found the enclosing radius of the nearest 4% of data points. If the radius was <8% or >30% of the arena diameter, then it was fixed at 8% or 30%, respectively. A Gaussian kernel set weights for each data point in this evaluation radius. For ratemaps, we computed weighted averages of trajectory data and spike data to create occupancy and spike density maps; dividing the spike density by the occupancy map produced the ratemap. For phase maps, we computed weighted mean resultant phase vectors from which we retrieved the mean phase and MVL. The mean phase across pixels produced the mean-phase maps; otherwise, the MVL was maximum-normalized and composited as a color saturation overlay onto the mean-phase map to produce the phase-vector map. Phase maps used colors drawn from the CIELUV color space to maintain perceptual uniformity of intensity across hues.

### Theta-rhythmic analysis

The rhythmicity index and burst-frequency estimates were derived from spike-timing autocorrelations. We adaptively smoothed 128-bin 0.5-s correlograms to find stable estimates of the first trough and first (non-central) peak of the correlograms. Rhythmicity was calculated as the ratio [(peak − trough)/peak]. Burst-frequency was calculated as the average of the first-peak mode estimate and an estimate based on a weighted-average of the first-to-second-trough correlations.

The theta modulation index was computed from a 10° binned phase histogram on [−π, π]. We circularly convolved the histogram with a 10° bandwidth Gaussian kernel for smoothing. Theta modulation was calculated as the ratio [(max − min)/max] of the smoothed histogram.

### Rate-phase regressions

We implemented the method of Kempter et al. (2012) [92] for computing circular-linear regressions with stable estimates of the correlation coefficient and *p*-value. This method was used for all rate-phase regression lines and rate-phase correlation values. For a given unit recording, the input data consisted of the common trajectory-sampled pixels from the 64 × 64-pixel ratemap and mean-phase map computed (as described above) from the unit’s spike data, LFP theta signal, and spatial trajectory. To compute the total phase shift, we multiplied the estimated rate-phase regression slope by the range of firing rates [max − min] in the ratemap.

### Stability analysis

We calculated spatial correlations as the mean-adjusted cosine vector similarity between the common trajectory-sampled pixels in 64 × 64-pixel ratemaps computed with the adaptive kernel (as described above). We calculated changes in total phase shift as the absolute difference between total phase shifts computed from rate-phase regressions on 64 × 64-pixel ratemaps and mean-phase maps. For the early-late within-session comparisons, the early portion consisted of up to 1-h after the start or the first half of the recording session data (whichever was shorter); the late portion consisted of up to 1-h before the end or the last half of the recording session data (whichever was shorter). The across-cell baseline consisted of each recording’s early portion paired with the late portion from every recording of all other identified cells. For the multiple-day comparisons, spatial correlations and changes in total phase shift were computed using the ratemaps and mean-phase maps based on the full recording session data (as in every analysis apart from the early-late comparisons). The within-cell comparison consisted of all unique pairs of a given cell’s recordings for all cells with multiple recordings. The across-cell baseline consisted of each recording from a cell with multiple recordings paired with every recording of all other identified cells.

### Information-theoretic measures

We computed spatial phase information *I*_phase_ as the mutual information between phase (*ϕ*) and position (*x*)

$$\begin{array}{c} I\left(\phi ;x\right)=\sum_{x}\sum_{\phi }p\left(\phi ,x\right)\log_{2}(\frac{p(\phi ,x)}{p(\phi )\;p(x)}) \end{array}$$

based on joint space-phase distributions of spikes binned into 15 × 15 × 36 (*x* × *y* × *ϕ*) arrays. This measure yielded information in units of bits. We permuted spike phases 1,000 times to calculate *p*-values.

We computed spike information content based on Skaggs’ formulation [45]

$$\begin{array}{c} I_{K}=\frac{1}{F}\sum_{k\in K}p\left(k\right)\;f\left(k\right)\log_{2}(\frac{f(k)}{F}) \end{array}$$

where *K* was position, direction, or speed of the trajectory; *p* was the occupancy density; *f* was a firing-rate function; and *F* was the mean firing rate. Position was binned into 15 × 15 arrays on [0, 80] cm along the *x* and *y* axes; direction into 36 bins on [0, 2π]; and speed into 18 bins on [5, 50] cm/s excluding bins with <3 s occupancy. These measures yielded information rates in units of bits/spike. We randomly shift-wrapped spike trains with 20-s minimum offsets and re-interpolated trajectory data 1,000 times to calculate *p*-values.

### Trajectory modulation

The direction modulation index was computed as the ratio [(max − min)/max] of a smoothed firing-rate function of movement direction. Average firing rates in 36 direction bins on [0, 2π] were circularly convolved with a 10° bandwidth Gaussian kernel. The speed modulation index was computed as the ratio [(max − min)/max] of a firing-rate function of speed. Average firing rates were calculated for 14 bins on [5, 40] cm/s excluding bins with <8 s occupancy.

### GLM training

Ridge regression models were trained on 9 scalar predictors representing the vector components of the 5 model variables: *L* = (*x*, *y*), *Q* = (*x*^2^, *y*^2^, *xy*), *W* (scalar), *S* (scalar), and *D* = (*u*_*x*_, *u*_*y*_). The wall predictor *W* was a sigmoid proximity signal [1/(1 + exp(−*k*(*r* − *w*_0_)))] for radius *r* from arena center, *k* = 0.5, and *w*_0_ = 30 cm. *S* was linear trajectory speed. *D* was the unit vector along the movement direction. Training samples were 300-ms bins and predictors were interpolated at the midpoint of each bin. Each predictor was standardized by subtracting its sample mean and dividing by its sample standard deviation. The response variable was the log spike-count *Y* for each bin, as in a Poisson-distributed GLM. The trajectory was divided into equal-sized 2 × 2 or 3 × 3 grids based on data limits. For each grid section, the GLM was trained on all data samples inside the section according to interpolated (*x*, *y*) position. Estimated model intercepts and coefficients for each recording and grid section were stored for analysis (or for the reduced LQW generative model). To regularize the model, tuning parameter *α* determined the *ℓ*_2_- norm penalty for least-squares optimization

$$\begin{array}{c} \hat{\beta }=\underset{\beta }{arg min}[\sum_{i=1}^{n_{t}}{\left(Y_{i}-{\hat{Y}}_{i}\right)}^{2}+\alpha {∥\beta ∥}_{2}^{2}] \end{array}$$

where *n*_*t*_ was the number of training samples. We maximized model directionality (or, similarly, the wall response *W* in the LQW generative model) by choosing

$$\begin{array}{c} \hat{\alpha }=\underset{\alpha }{arg max}[\frac{1}{n_{r}}\sum_{k=1}^{n_{r}}\frac{e^{{∥\beta_{D,k}∥}_{2}}\cdot n_{t,k}}{\sum_{j\in \{LQWSD\}}e^{{∥\beta_{j,k}∥}_{2}}\sum_{i}{\left(K_{i,k}-{\hat{K}}_{i,k}\right)}^{2}}] \end{array}$$ (14)

which maximizes (over *n*_*r*_ = 1, 073 single-unit recordings) the softmax directional coefficients while minimizing spike-count (*K* = exp(*Y*)) prediction errors (MSE; S4 Fig). The value *α* = 1.2496 from the 2 × 2 model was used for analysis because of higher likelihood, lower MSE, lower penalty, and complete wall contact across grid sections compared to the 3 × 3 model.

### GLM analysis

The relative strengths of GLM variables were computed as normalized vector norms

$$\begin{array}{c} \text{Strength}\left(X\right)=\frac{\sum_{i=1}^{g}{∥\beta_{X}^{i}∥}_{2}^{2}}{\sum_{j\in \{LQWSD\}}\sum_{i=1}^{g}{∥\beta_{j}^{i}∥}_{2}^{2}} \end{array}$$ (15)

for variable *X* ∈ {*L*, *Q*, *W*, *S*, *D*} across *g* grid sections. Thus DSI was computed as Strength(*D*) and DHI was computed as 1 minus the angular s.d. of the *β*_*D*_ vectors across the grid. The maximal contributions of GLM variables were computed similarly to Eq (15) but with maximum linear predictors (Eq (2)) instead of coefficient vector norms. The sum across variables for both relative strength and maximal contribution was normalized within recordings and then averaged by unique cell (Fig 5). Grid matrix plots (S6 Fig, panel A+C) show these values prior to the grid summations (Eq (15)).

To reconstruct ratemaps, we used the midpoints of grid-specific training samples to predict spike counts from the model for each grid section. We collated the counts and sample positions across grid sections to reconstitute a complete dataset for generating the ratemap.

To create the LQW generative model, we used a COBYLA search to find the arena-bounded minimum and maximum of the linear predictor for each recording. We normalized the LQW parameters to [0, 1] and applied a clipping sigmoid [1/(1 + exp(−10(*f* − 0.5)))] to smoothly enforce the range of the resulting spatial function. To sample the generative model, we randomly selected a negative phaser’s spatial function, added 20% Gaussian noise to its LQW parameters, and rotated the function about the center by a random angle.

### Software

Data analysis and modeling were conducted using custom python packages that depend on libraries from the open-source ecosystem: numpy, scipy, matplotlib, seaborn, pandas, scikit-learn, pytables, Brian2, and others. The source code, including a complete specification of the python environment, is available at doi.org/10.6084/m9.figshare.6072317.

## Supporting information

S1 Fig Spatial phase-coding cells were theta-modulated and theta-rhythmic. We show distributions of single-unit recordings with non-significant spatial phase information *I*_phase_ (‘non-phase-coding’, n.s., orange; *n* = 840) or significant *I*_phase_ (‘phase-coding’, *p* < 0.02, blue; *n* = 233; Methods). Violin plots show Gaussian kernel-density estimates (using Scott’s bandwidth rule) normalized by group size for each split; long-dash lines, medians; short-dash lines, 1st/3rd quartiles. (A) Phase-coding recordings had maximal spatial firing rates (median, 7.35 spikes/s) that were distributed higher than non-phase-coding recordings. (B) Autocorrelogram-based estimates of burst frequency (Methods) were similar (median: phase-coding, 7.66 s^−1^; non-phase-coding, 7.65), but phase-coding recordings were more narrowly distributed (interquartile range: 0.524) than non-phase-coding recordings (1.031). (C) Theta modulation and rhythmicity indices (Methods) show that phase-coding recordings were distributed higher, but this is likely due to the substantial low-rhythmicity subpopulation evident in non-phase-coding recordings. Jittered strip plots show every phase-coding data point. (D+E) Spatial phase-coding cells had broadly distributed rate-phase correlations. (D) *I*_phase_ for phase-coding cells (median, 0.36 bits) was positively skewed across a wide range ([0.012, 3.67]bits). (E) Circular-linear regressions of mean phase onto mean rate based on spatial map pixels. Non-phase-coding recordings were distributed around zero. Correlation coefficient (left) and total phase shift (right; Methods) showed broader distributions for phase-coding than non-phase-coding cells: Compare quartiles (short-dash lines) and fatter tails reflecting excess negative and positive correlations. Total phase shift (right) was computed by rate-normalizing the regression slope (middle).
(PDF)

S2 Fig Phaser cells: Moderate firing rates and stable spatial phase coding. (A) Violin plots show distributions comparing spatial phase-coding recordings (with significant *I*_phase_) that were not selected (‘nonphaser’; *n* = 233) or were selected (‘phaser’; *n* = 101) by the phaser cell criteria (see numbered listing of criteria preceding Fig 2 in Results). (Left) Maximal spatial firing rates for phaser cell recordings had a substantially restricted range (interquartile interval, [5.34, 9.86] s^−1^) compared to nonphaser recordings ([2.94, 20.4]). Note, a minimum firing rate of 3.5 spikes/s was one of the phaser cell criteria, and the *y*-axis truncates, for clarity, nonphaser data that is shown in S1 Fig, panel A. The observed range is commensurate with activity that, on average, consists of 1 or 2 spikes per theta cycle at theta frequencies from 5–12 Hz. Theoretically, having fewer spikes per theta cycle decreases the lower bound of spike-phase variance, which may enhance the effectiveness of temporal coding by oscillatory phase. (Right) Theta rhythmicity of phaser cell recordings was distributed similarly, but slightly lower than nonphaser cell recordings. (B) Phaser cells recorded across multiple days (*n* = 19) demonstrated substantial stability in day-to-day measurements of phase-coding quantities: spatial phase information (left) and total phase shift (right). Large jumps (or sign-changing for phase shifts) were relatively rare (3/19 cells). The phase shift data (right) is the basis for the within-cell pair-wise phase-coding histogram in Fig 2E. Only phaser-classified recordings for each cell are shown. Lines are color-coded to unique cells.
(PDF)

S3 Fig Anatomical distribution and space–trajectory coding of phaser cell recordings. (A) Counts of uniquely identified cells with at least one negative or positive phaser-classified recording. (Left) Distributions of recorded phaser cell locations across brain areas. Hipp. = hippocampus; Thal. = thalamus; Other includes nucleus accumbens, caudate nucleus, and putamen. (Right) Distribution across septal recording sites. IG = indusium griseum; LS = lateral septum; LSD = dorsal nucleus of the lateral septum; LSI = intermediate nucleus of the lateral septum; Ld = lambdoid septal zone; SHi = septal-hippocampal nucleus; UNK = unknown; gcc = genu of the corpus callosum. (B) Comparison of spatial phase information *I*_phase_ with spike information content (Methods; [45]) for position (‘spatial rate information’; left), direction (middle), and speed (right). Most phaser cells carried strong spatial rate information (left) and a minority carried relatively low direction (middle) or speed (right) information. Stars: hippocampal (hipp.) recordings; circles: non-hippocampal (not hipp.) recordings; dashed lines: parity; solid lines: least-squares optimized slopes. (C) Trajectory-based firing-rate modulation indices (Methods) revealed potential source of bias in spatial recordings. Histograms: modulation indices for direction (left) and speed (right), positive data composited over negative. Gray line: kernel-density estimate (0.05 bandwidth Gaussian) of nonphaser cell recordings (arbitrary scale for visual comparison).
(PDF)

S4 Fig Regularization and shrinkage curves used for training GLM models. We trained GLMs to predict spike counts in 300-ms intervals based on spatial (*L*, *Q*, *W*) and/or trajectory-based (*S*, *D*) variables (Methods). For the analysis (Fig 5B+5C; S6 Fig), the model was trained and tested on a 3 × 3 spatial grid (C); however, the penalty parameter used for training was derived by optimizing the model on a 2 × 2 grid (B). Both values were similar, but the 2 × 2 value (B, bottom) was used because the directional likelihood was strongly peaked and the model better captured wall responses because the center grid of the 3 × 3 model was isolated from the walls. The GLM that we used to generate spatial inputs for the realistic 2D open-field phaser simulations was trained only on the spatial variables (A, 1 × 1 grid). (Top) Absolute model weights for each variable. (Second row) Softmax normalization of absolute model weights. (Third row) Spike-count prediction errors. (Last row) Model likelihood is the softmax *W* (A) or *D* (B+C) divided by the prediction error (Eq (14); Methods). The maximum likelihood *α* parameter (red circle) was chosen as the *ℓ*_2_- regularization penalty for the ridge regressions.
(PDF)

S5 Fig GLM ratemap reconstructions for example directional cells. To show that the LQW-SD 3 × 3 model could accurately reconstruct ratemaps of directional cells, we show example cells with homogeneous (A) and inhomogeneous (B) directionality. (A) The high maximal firing rates and crescent-like spatial modulation indicate that these may have been head-direction cells or cells with head-direction inputs. The GLM’s directional predictors (arrows) were consistently large and well-aligned across grid sections. (B) Recordings with inhomogeneous directionality showed minimal spatial modulation but included center-facing (left) and clockwise (middle) or anti-clockwise (right) directionality.
(PDF)

S6 Fig GLM weights and contributions for every phaser cell recording. GLM weights (A+B) and maximal contributions (C-E) for phaser cell recordings are shown in pseudocolor matrix plots. For visualization, recordings are presented in the same order in every grid section and grid average according to the expected value of the cell’s grid-averaged model weights to the left (toward *L*, i.e., more spatial) or right (toward *D*, i.e., more trajectory-related). To reveal model structure, each variable row in a grid section was sum-normalized and the paired grid plots (A+B, C+D) share color scales. (E) The contribution averages from (D) are displayed by phaser cell subtype: negative (left) and positive (right). The two subtypes demonstrated qualitatively similar inverse patterns of spatial (*L*, *Q*) vs. speed-related (*S*) contributions to firing.
(PDF)

S7 Fig Noisy theta-bursting target neuron model: Pulse synchronization. An intrinsic bursting model (Eq (11); [51]) was tuned with constant input (Table 4) to fire doublet bursts (A) close to the reference theta frequency, 7.5 Hz. The deviation between the reference frequency and the resulting burst rate, 7.519 bursts/s, meant that the unit’s theta phase (B) slowly drifted (precessed) over time (gray line). To test whether this unit could be phase-synchronized by periodic stimulation, we simulated an instantaneous pulse (*V* ← *V*+ 15mV) every other theta cycle at theta peak (0 radians). This pulse-synchronized unit (B, orange line) monotonically delayed toward theta peak and then (around 5 s into the simulation) discontinuously jumped past theta peak before slowly precessing to just before the peak. This dynamic, of jumping forward and precessing back, repeated (around 9 s) and continued stereotypically. This sawtooth pattern encapsulated the model’s theta-synchronization dynamics. For simulations with phaser network input, we added a stochastic input current to this ‘target burster’ model (Eq (11)). We chose a noise level (Table 4) that preserved theta bursting (C, same as Fig 7C, inset) but caused its burst phase to randomly drift over a 30-s simulation (D, gray dots, 36 trials). With noise, the pulse stimulation was able to reproduce the sawtooth pattern of synchronization (D, orange line).
(PDF)

S8 Fig 1D phaser-target entrainment across noise and phaser input levels. We show additional 1-hr simulations of the 1D phaser-target network shown in Fig 7F+7G. (A) With the input gain from the phasers fixed (Table 4), simulations with 0.0*σ*, 0.1*σ*, 0.3*σ*, and 0.5*σ* noise levels demonstrated that the supervised modes of the artificial phase-code remained functional across different levels of noise. (B) With the noise level fixed at 0.3*σ*, the effect of zero phaser input gain (top left) can be compared to weaker (top right) and stronger (bottom right) levels of phaser input. Weak phaser input (top right) entrained the target burster, but the phase trajectories were extended due to the slower development of phase locking on approaches toward positions 0 or 1.
(PDF)

S9 Fig Generative samples of model LQW-phasers in open-field simulations. (A) Ratemap/phase-map pairs are shown for 50/1,000 negative phasers from the realistic 2D open-field simulations (Fig 8). The rate and phase response of each phaser was driven by a randomly sampled spatial function from the LQW generative input model (S10 Fig, panel A). In the phase maps, note that the phasers advanced from pre-theta-peak (green; see phase-vector color wheel at bottom) to theta-trough (pink) from low- to high-rate regions. Missing phase map pixels reflect insufficient numbers of nearby spikes for spatial averaging. (B) Ratemap/phase-map pairs are shown for 50/1,000 positive phasers. The rate and phase response of each phaser was driven by theta excitation and feedforward inhibition from a negative phaser with an LQW-generated spatial input (A). In the phase maps, note that the phasers delayed from theta peak (green) to halfway through the falling phase (blue/green; π/2 radians). Like the 1D model (Fig 6) and phaser cell recordings (Fig 4), the positive rate-phase coupling was weaker than the negative.
(PDF)

S10 Fig Bayesian decoding of target burst phase from open-field simulations. Realistic 2D simulations of phasers and target neurons were simulated and the bursting activity of the target neurons was decoded to assess position-error correction (Methods). (A) The steps to sample spatial input functions from the generative model for negative phasers are illustrated (Methods). From left to right: Phaser cell recordings (examples from Fig 4A) were learned by the 1 × 1 LQW model (Eq (3)) and their linear predictor functions were normalized to [0, 1] with a sigmoid nonlinearity. To generate a novel spatial input, we randomly selected one of these normalized spatial functions, added 20% Gaussian noise to the LQW parameters, and randomly center-rotated the coordinate frame. (B) Target networks were simple collections of target burster units. The Ring collection of target bursters varied across phase offsets (orange); the Phase 1 and Phase 2 collections varied across preferred direction at opposing phase offsets (blue and green). (C) Normalized temporal autocorrelograms of decoding error for full-sized collections (64 units in each collection; 192 units for the combination of all collections). The correlation width indicates the timescale of error correction, which was quantified as the HWHM timescale in Fig 8H (Methods).
(PDF)

S1 Movie Competitive 2D open-field phaser entrainment across spatial phase offsets. The spatial phase codes in Fig 8B differed by the reference phase offset of the VCO-like phase code. Here we show a movie in which the frames iterate through 64 units in the Ring collection of target bursters (S10 Fig, panel B, orange) that were simulated with a 600-s behavioral trajectory. The supervised phase code (top left) moves smoothly along the 45° diagonal for a complete cycle, allowing the video to be looped. The broad negative/positive (pink/blue) synchronization regions competed to encode the environment for each of the different target bursters in the collection. (top right) Space-phase distribution of the total phaser network input to the target burster. (bottom left) Burst phase map of target burster output.
(MP4)

S2 Movie Competitive 2D open-field phaser entrainment across preferred direction: Phase 1. The spatial phase codes in Fig 8B have a 45° preferred direction, which determines the angular orientation of the VCO-like phase code. Here we show a movie in which the frames iterate through 64 units in the Phase 1 collection of target bursters (S10 Fig, panel B, blue) that were simulated with a 600-s behavioral trajectory. The supervised phase code (top left) rotates smoothly for a complete cycle, allowing the video to be looped. With this phase offset (0.0, at the center of the arena), the negative phasers synchronized a boundary region (oranges/pinks) along the preferred direction. (top right) Space-phase distribution of the total phaser network input to the target burster. (bottom left) Burst phase map of target burster output.
(MP4)

S3 Movie Competitive 2D open-field phaser entrainment across preferred direction: Phase 2. The spatial phase codes in Fig 8B have a 45° preferred direction, which determines the angular orientation of the VCO-like phase code. Here we show a movie in which the frames iterate through 64 units in the Phase 2 collection of target bursters (S10 Fig, panel B, green) that were simulated with a 600-s behavioral trajectory. The supervised phase code (top left) rotates smoothly for a complete cycle, allowing the video to be looped. With this phase offset (π, at the center of the arena), the positive phasers synchronized a boundary region (blue/green) along the preferred direction. (top right) Space-phase distribution of the total phaser network input to the target burster. (bottom left) Burst phase map of target burster output.
(MP4)
