# Supplementary material for: Spatial synchronization codes from coupled rate-phase neurons
Source: PLoS Comput Biol. 2019 Jan 25;15(1):e1006741. doi: 10.1371/journal.pcbi.1006741 (PMC6364943; doi:10.1371/journal.pcbi.1006741)
Supplement: S1 Fig — We show distributions of single-unit recordings with non-significant spatial phase information Iphase (‘non-phase-coding’, n.s., orange; n = 840) or significant Iphase (‘phase-coding’, p < 0.02, blue; n = 233; Methods). Violin plots show Gaussian kernel-density estimates (using Scott’s bandwidth rule) normalized by group size for each split; long-dash lines, medians; short-dash lines, 1st/3rd quartiles. (A) Phase-coding recordings had maximal spatial firing rates (median, 7.35 spikes/s) that were distributed higher than non-phase-coding recordings. (B) Autocorrelogram-based estimates of burst frequency (Methods) were similar (median: phase-coding, 7.66 s−1; non-phase-coding, 7.65), but phase-coding recordings were more narrowly distributed (interquartile range: 0.524) than non-phase-coding recordings (1.031). (C) Theta modulation and rhythmicity indices (Methods) show that phase-coding recordings were distributed higher, but this is likely due to the substantial low-rhythmicity subpopulation evident in non-phase-coding recordings. Jittered strip plots show every phase-coding data point. (D+E) Spatial phase-coding cells had broadly distributed rate-phase correlations. (D) Iphase for phase-coding cells (median, 0.36 bits) was positively skewed across a wide range ([0.012, 3.67]bits). (E) Circular-linear regressions of mean phase onto mean rate based on spatial map pixels. Non-phase-coding recordings were distributed around zero. Correlation coefficient (left) and total phase shift (right; Methods) showed broader distributions for phase-coding than non-phase-coding cells: Compare quartiles (short-dash lines) and fatter tails reflecting excess negative and positive correlations. Total phase shift (right) was computed by rate-normalizing the regression slope (middle). (PDF) [file pcbi.1006741.s001.pdf]

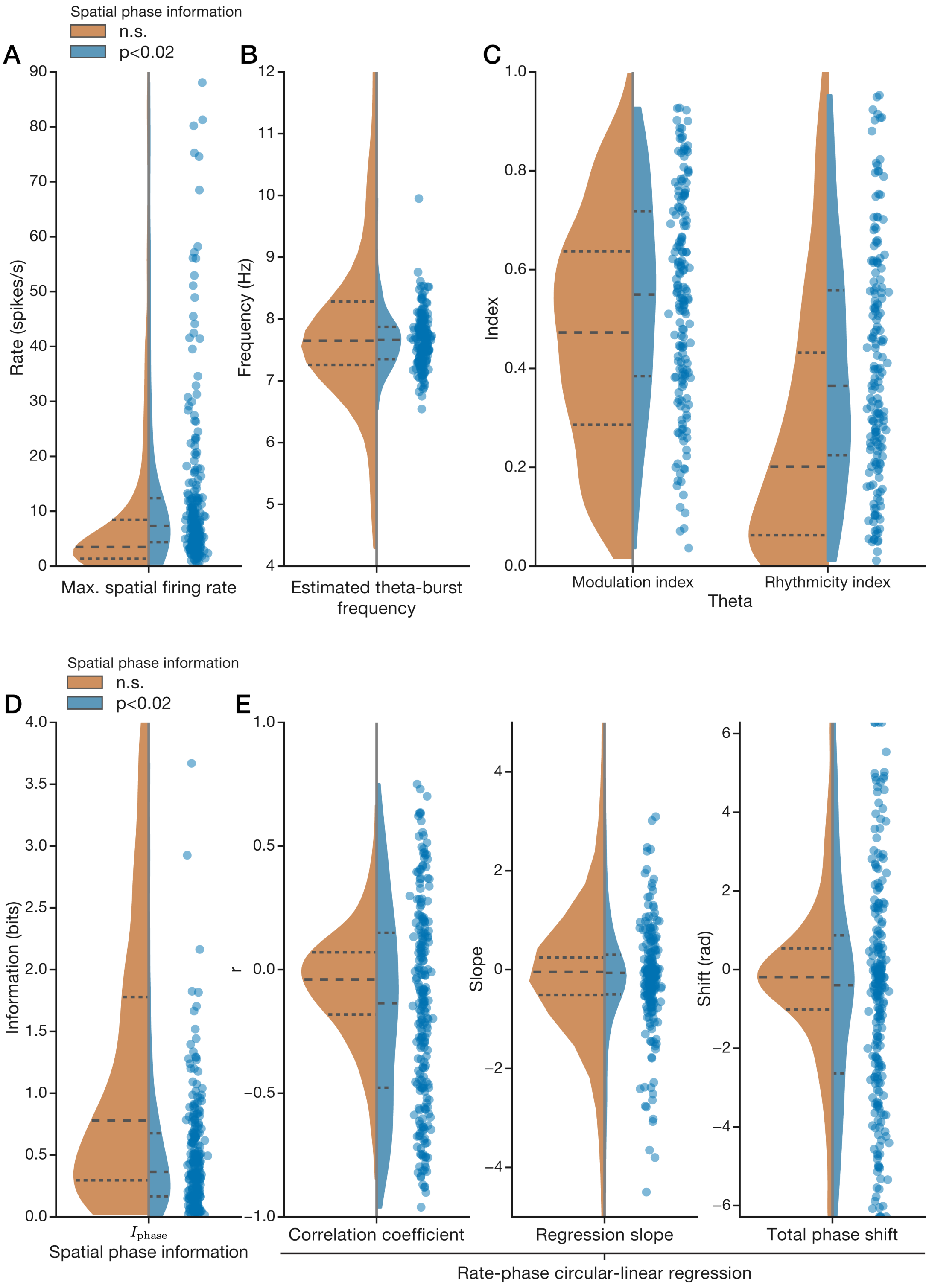

S1 Fig. *See legend on reverse.*

S1 Fig. Spatial phase-coding cells were theta-modulated and theta-rhythmic. We show distributions of single-unit recordings with non-significant spatial phase information I_{phase} ('non-phase-coding', n.s., orange; $n = 840$) or significant I_{phase} ('phase-coding', $p < 0.02$, blue; $n = 233$; Methods). Violin plots show Gaussian kernel-density estimates (using Scott's bandwidth rule) normalized by group size for each split; long-dash lines, medians; short-dash lines, 1st/3rd quartiles. (A) Phase-coding recordings had maximal spatial firing rates (median, 7.35 spikes/s) that were distributed higher than non-phase-coding recordings. (B) Autocorrelogram-based estimates of burst frequency (Methods) were similar (median: phase-coding, $7.66\,\text{s}^{-1}$; non-phase-coding, 7.65), but phase-coding recordings were more narrowly distributed (interquartile range: 0.524) than non-phase-coding recordings (1.031). (C) Theta modulation and rhythmicity indices (Methods) show that phase-coding recordings were distributed higher, but this is likely due to the substantial low-rhythmicity subpopulation evident in non-phase-coding recordings. Jittered strip plots show every phase-coding data point. (D+E) Spatial phase-coding cells had broadly distributed rate-phase correlations. (D) I_{phase} for phase-coding cells (median, 0.36 bits) was positively skewed across a wide range ($[0.012, 3.67]$ bits). (E) Circular-linear regressions of mean phase onto mean rate based on spatial map pixels. Non-phase-coding recordings were distributed around zero. Correlation coefficient (left) and total phase shift (right; Methods) showed broader distributions for phase-coding than non-phase-coding cells: Compare quartiles (short-dash lines) and fatter tails reflecting excess negative and positive correlations. Total phase shift (right) was computed by rate-normalizing the regression slope (middle).
