# Supplementary material for: Spatial synchronization codes from coupled rate-phase neurons
Source: PLoS Comput Biol. 2019 Jan 25;15(1):e1006741. doi: 10.1371/journal.pcbi.1006741 (PMC6364943; doi:10.1371/journal.pcbi.1006741)
Supplement: S2 Fig — (A) Violin plots show distributions comparing spatial phase-coding recordings (with significant Iphase) that were not selected (‘nonphaser’; n = 233) or were selected (‘phaser’; n = 101) by the phaser cell criteria (see numbered listing of criteria preceding Fig 2 in Results). (Left) Maximal spatial firing rates for phaser cell recordings had a substantially restricted range (interquartile interval, [5.34, 9.86] s−1) compared to nonphaser recordings ([2.94, 20.4]). Note, a minimum firing rate of 3.5 spikes/s was one of the phaser cell criteria, and the y-axis truncates, for clarity, nonphaser data that is shown in S1 Fig, panel A. The observed range is commensurate with activity that, on average, consists of 1 or 2 spikes per theta cycle at theta frequencies from 5–12 Hz. Theoretically, having fewer spikes per theta cycle decreases the lower bound of spike-phase variance, which may enhance the effectiveness of temporal coding by oscillatory phase. (Right) Theta rhythmicity of phaser cell recordings was distributed similarly, but slightly lower than nonphaser cell recordings. (B) Phaser cells recorded across multiple days (n = 19) demonstrated substantial stability in day-to-day measurements of phase-coding quantities: spatial phase information (left) and total phase shift (right). Large jumps (or sign-changing for phase shifts) were relatively rare (3/19 cells). The phase shift data (right) is the basis for the within-cell pair-wise phase-coding histogram in Fig 2E. Only phaser-classified recordings for each cell are shown. Lines are color-coded to unique cells. (PDF) [file pcbi.1006741.s002.pdf]

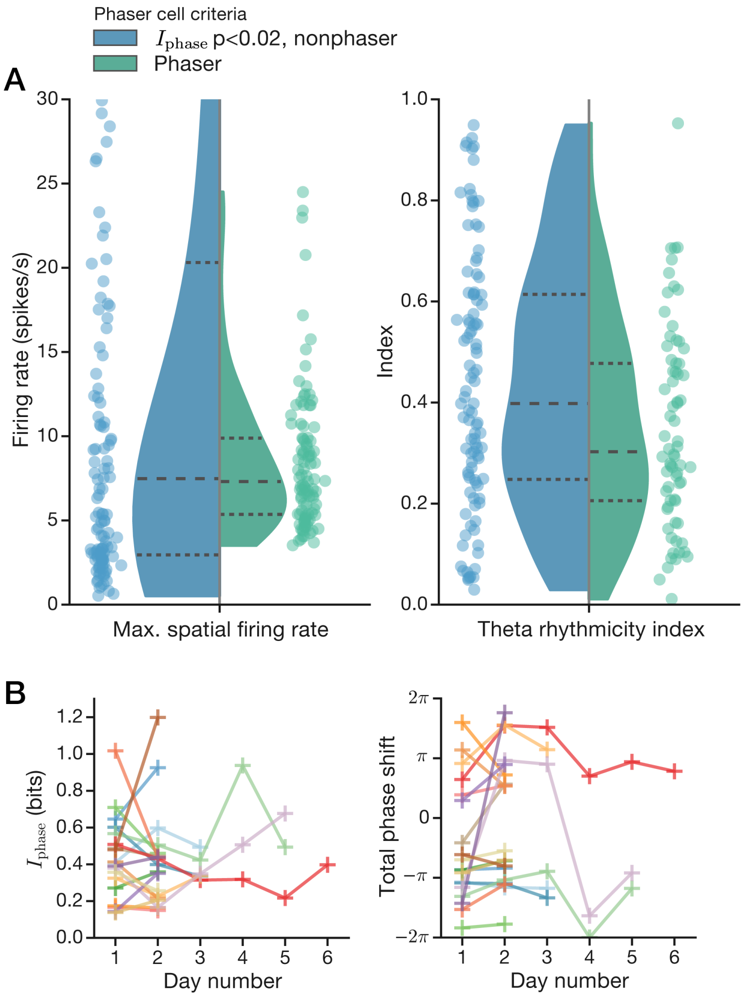

S2 Fig. Phaser cells: moderate firing rates and stable spatial phase coding. (A) Violin plots show distributions comparing spatial phase-coding recordings (with significant I_{phase}) that were not selected ('nonphaser'; $n = 233$) or were selected ('phaser'; $n = 101$) by the phaser cell criteria (see numbered listing of criteria preceding Fig 2 in Results). (Left) Maximal spatial firing rates for phaser cell recordings had a substantially restricted range (interquartile interval, [5.34, 9.86] s^{-1}) compared to nonphaser recordings ([2.94, 20.4]). Note, a minimum firing rate of 3.5 spikes/s was one of the phaser cell criteria, and the y-axis truncates, for clarity, nonphaser data that is shown in , panel A. The observed range is commensurate with activity that, on average, consists of 1 or 2 spikes per theta cycle at theta frequencies from 5–12 Hz. Theoretically, having fewer spikes per theta cycle decreases the lower bound of spike-phase variance, which may enhance the effectiveness of temporal coding by oscillatory phase. (Right) Theta rhythmicity of phaser cell recordings was distributed similarly, but slightly lower than nonphaser cell recordings. (B) Phaser cells recorded across multiple days ($n = 19$) demonstrated substantial stability in day-to-day measurements of phase-coding quantities: spatial phase information (left) and total phase shift (right). Large jumps (or sign-changing for phase shifts) were relatively rare (3/19 cells). The phase shift data (right) is the basis for the within-cell pair-wise phase-coding histogram in Fig 2E. Only phaser-classified recordings for each cell are shown. Lines are color-coded to unique cells.
