# Supplementary material for: Spatial synchronization codes from coupled rate-phase neurons
Source: PLoS Comput Biol. 2019 Jan 25;15(1):e1006741. doi: 10.1371/journal.pcbi.1006741 (PMC6364943; doi:10.1371/journal.pcbi.1006741)
Supplement: S3 Fig — (A) Counts of uniquely identified cells with at least one negative or positive phaser-classified recording. (Left) Distributions of recorded phaser cell locations across brain areas. Hipp. = hippocampus; Thal. = thalamus; Other includes nucleus accumbens, caudate nucleus, and putamen. (Right) Distribution across septal recording sites. IG = indusium griseum; LS = lateral septum; LSD = dorsal nucleus of the lateral septum; LSI = intermediate nucleus of the lateral septum; Ld = lambdoid septal zone; SHi = septal-hippocampal nucleus; UNK = unknown; gcc = genu of the corpus callosum. (B) Comparison of spatial phase information Iphase with spike information content (Methods; [45]) for position (‘spatial rate information’; left), direction (middle), and speed (right). Most phaser cells carried strong spatial rate information (left) and a minority carried relatively low direction (middle) or speed (right) information. Stars: hippocampal (hipp.) recordings; circles: non-hippocampal (not hipp.) recordings; dashed lines: parity; solid lines: least-squares optimized slopes. (C) Trajectory-based firing-rate modulation indices (Methods) revealed potential source of bias in spatial recordings. Histograms: modulation indices for direction (left) and speed (right), positive data composited over negative. Gray line: kernel-density estimate (0.05 bandwidth Gaussian) of nonphaser cell recordings (arbitrary scale for visual comparison). (PDF) [file pcbi.1006741.s003.pdf]

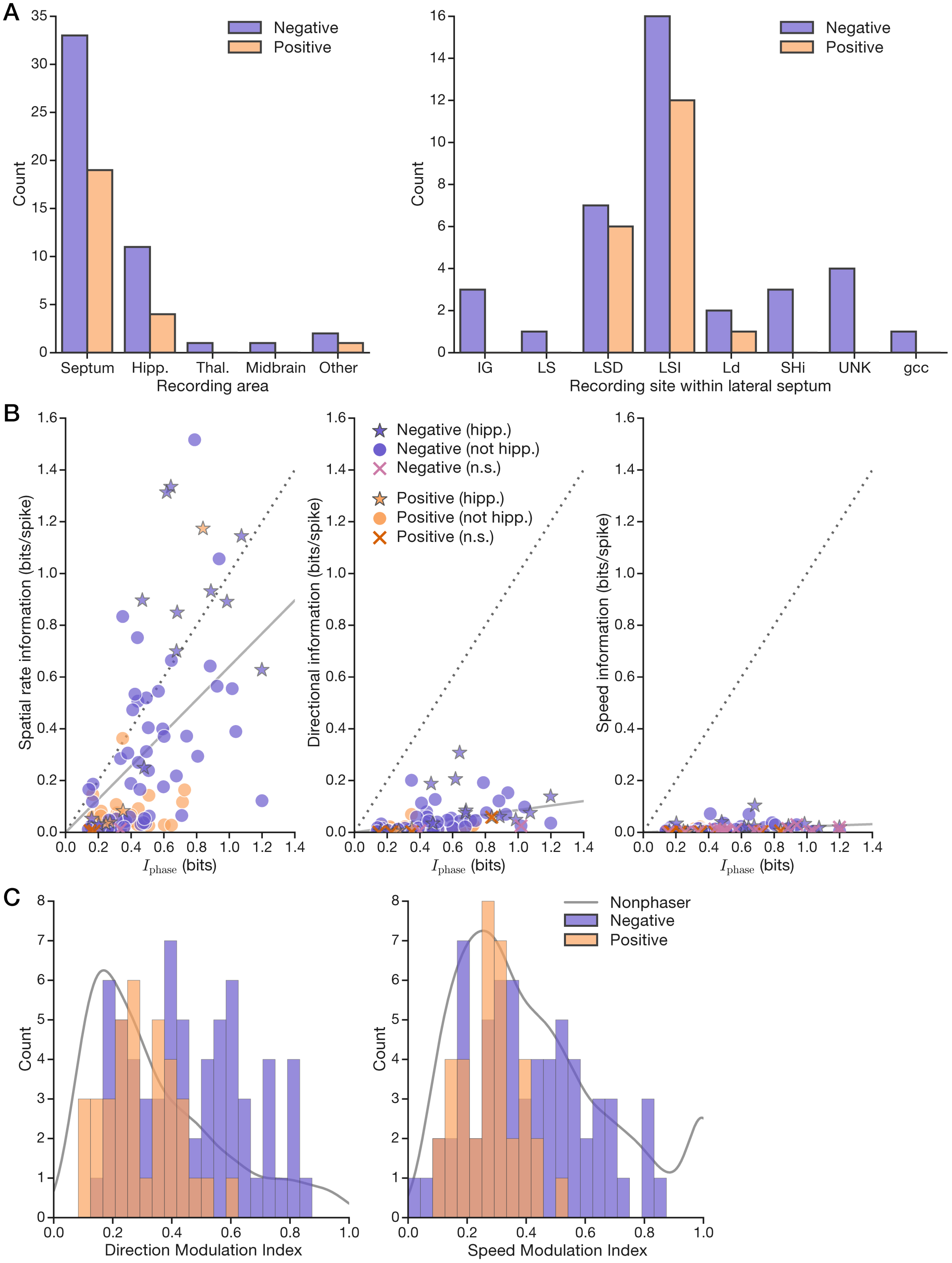

S3 Fig. *See legend on reverse.*

S3 Fig. Anatomical distribution and space–trajectory coding of phaser cell recordings. (A) Counts of uniquely identified cells with at least one negative or positive phaser-classified recording. (Left) Distributions of recorded phaser cell locations across brain areas. Hipp. = hippocampus; Thal. = thalamus; Other includes nucleus accumbens, caudate nucleus, and putamen. (Right) Distribution across septal recording sites. IG = indusium griseum; LS = lateral septum; LSD = dorsal nucleus of the lateral septum; LSI = intermediate nucleus of the lateral septum; Ld = lambdoid septal zone; SHi = septal-hippocampal nucleus; UNK = unknown; gcc = genu of the corpus callosum. (B) Comparison of spatial phase information I_{phase} with spike information content (Methods; [45]) for position ('spatial rate information'; left), direction (middle), and speed (right). Most phaser cells carried strong spatial rate information (left) and a minority carried relatively low direction (middle) or speed (right) information. Stars: hippocampal (hipp.) recordings; circles: non-hippocampal (not hipp.) recordings; dashed lines: parity; solid lines: least-squares optimized slopes. (C) Trajectory-based firing-rate modulation indices (Methods) revealed potential source of bias in spatial recordings. Histograms: modulation indices for direction (left) and speed (right), positive data composited over negative. Gray line: kernel-density estimate (0.05 bandwidth Gaussian) of nonphaser cell recordings (arbitrary scale for visual comparison).
