# Supplementary material for: Spatial synchronization codes from coupled rate-phase neurons
Source: PLoS Comput Biol. 2019 Jan 25;15(1):e1006741. doi: 10.1371/journal.pcbi.1006741 (PMC6364943; doi:10.1371/journal.pcbi.1006741)
Supplement: S4 Fig — We trained GLMs to predict spike counts in 300-ms intervals based on spatial (L, Q, W) and/or trajectory-based (S, D) variables (Methods). For the analysis (Fig 5B+5C; S6 Fig), the model was trained and tested on a 3 × 3 spatial grid (C); however, the penalty parameter used for training was derived by optimizing the model on a 2 × 2 grid (B). Both values were similar, but the 2 × 2 value (B, bottom) was used because the directional likelihood was strongly peaked and the model better captured wall responses because the center grid of the 3 × 3 model was isolated from the walls. The GLM that we used to generate spatial inputs for the realistic 2D open-field phaser simulations was trained only on the spatial variables (A, 1 × 1 grid). (Top) Absolute model weights for each variable. (Second row) Softmax normalization of absolute model weights. (Third row) Spike-count prediction errors. (Last row) Model likelihood is the softmax W (A) or D (B+C) divided by the prediction error (Eq (14); Methods). The maximum likelihood α parameter (red circle) was chosen as the ℓ2- regularization penalty for the ridge regressions. (PDF) [file pcbi.1006741.s004.pdf]

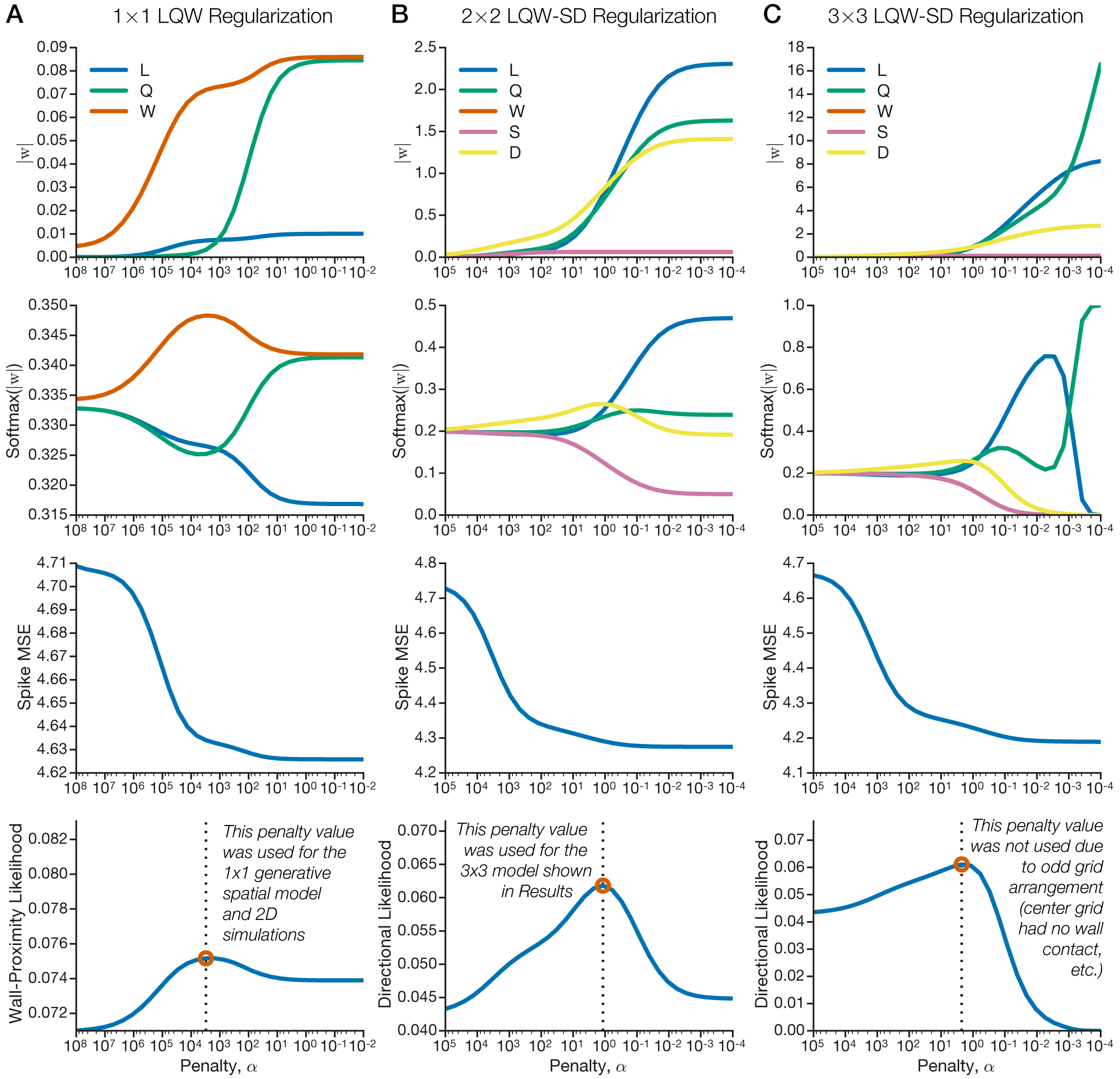

S4 Fig. *See legend on reverse.*

S4 Fig. Regularization and shrinkage curves used for training GLM models. We trained GLMs to predict spike counts in 300-ms intervals based on spatial (L, Q, W) and/or trajectory-based (S, D) variables (Methods). For the analysis (Fig 5B+C; S4 Fig, the model was trained and tested on a 3×3 spatial grid (C); however, the penalty parameter used for training was derived by optimizing the model on a 2×2 grid (B). Both values were similar, but the 2×2 value (B, bottom) was used because the directional likelihood was strongly peaked and the model better captured wall responses because the center grid of the 3×3 model was isolated from the walls. The GLM that we used to generate spatial inputs for the realistic 2D open-field phaser simulations was trained only on the spatial variables (A, 1×1 grid). (Top) Absolute model weights for each variable. (Second row) Softmax normalization of absolute model weights. (Third row) Spike-count prediction errors. (Last row) Model likelihood is the softmax W (A) or D (B+C) divided by the prediction error (Eq (14); Methods). The maximum likelihood α parameter (red circle) was chosen as the ℓ_2-regularization penalty for the ridge regressions.
