# Supplementary material for: Spatial synchronization codes from coupled rate-phase neurons
Source: PLoS Comput Biol. 2019 Jan 25;15(1):e1006741. doi: 10.1371/journal.pcbi.1006741 (PMC6364943; doi:10.1371/journal.pcbi.1006741)
Supplement: S5 Fig — To show that the LQW-SD 3 × 3 model could accurately reconstruct ratemaps of directional cells, we show example cells with homogeneous (A) and inhomogeneous (B) directionality. (A) The high maximal firing rates and crescent-like spatial modulation indicate that these may have been head-direction cells or cells with head-direction inputs. The GLM’s directional predictors (arrows) were consistently large and well-aligned across grid sections. (B) Recordings with inhomogeneous directionality showed minimal spatial modulation but included center-facing (left) and clockwise (middle) or anti-clockwise (right) directionality. (PDF) [file pcbi.1006741.s005.pdf]

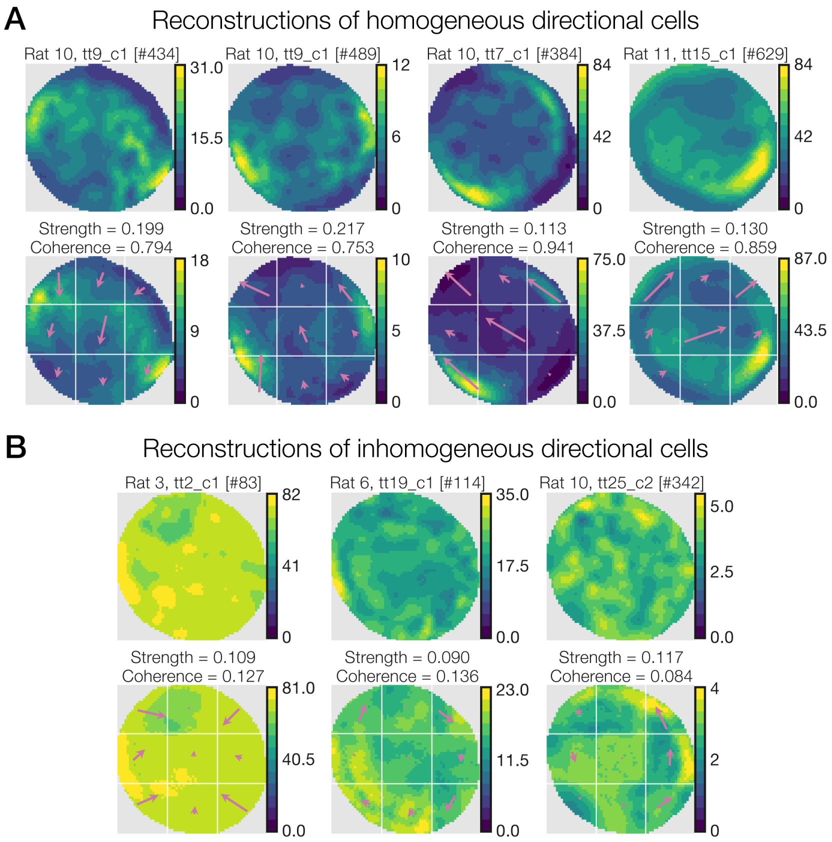

S5 Fig. GLM ratemap reconstructions for example directional cells. To show that the LQW-SD 3×3 model could accurately reconstruct ratemaps of directional cells, we show example cells with homogeneous (A) and inhomogeneous (B) directionality. (A) The high maximal firing rates and crescent-like spatial modulation indicate that these may have been head-direction cells or cells with head-direction inputs. The GLM's directional predictors (arrows) were consistently large and well-aligned across grid sections. (B) Recordings with inhomogeneous directionality showed minimal spatial modulation but included center-facing (left) and clockwise (middle) or anti-clockwise (right) directionality.
