# Supplementary material for: Spatial synchronization codes from coupled rate-phase neurons
Source: PLoS Comput Biol. 2019 Jan 25;15(1):e1006741. doi: 10.1371/journal.pcbi.1006741 (PMC6364943; doi:10.1371/journal.pcbi.1006741)
Supplement: S6 Fig — GLM weights (A+B) and maximal contributions (C-E) for phaser cell recordings are shown in pseudocolor matrix plots. For visualization, recordings are presented in the same order in every grid section and grid average according to the expected value of the cell’s grid-averaged model weights to the left (toward L, i.e., more spatial) or right (toward D, i.e., more trajectory-related). To reveal model structure, each variable row in a grid section was sum-normalized and the paired grid plots (A+B, C+D) share color scales. (E) The contribution averages from (D) are displayed by phaser cell subtype: negative (left) and positive (right). The two subtypes demonstrated qualitatively similar inverse patterns of spatial (L, Q) vs. speed-related (S) contributions to firing. (PDF) [file pcbi.1006741.s006.pdf]

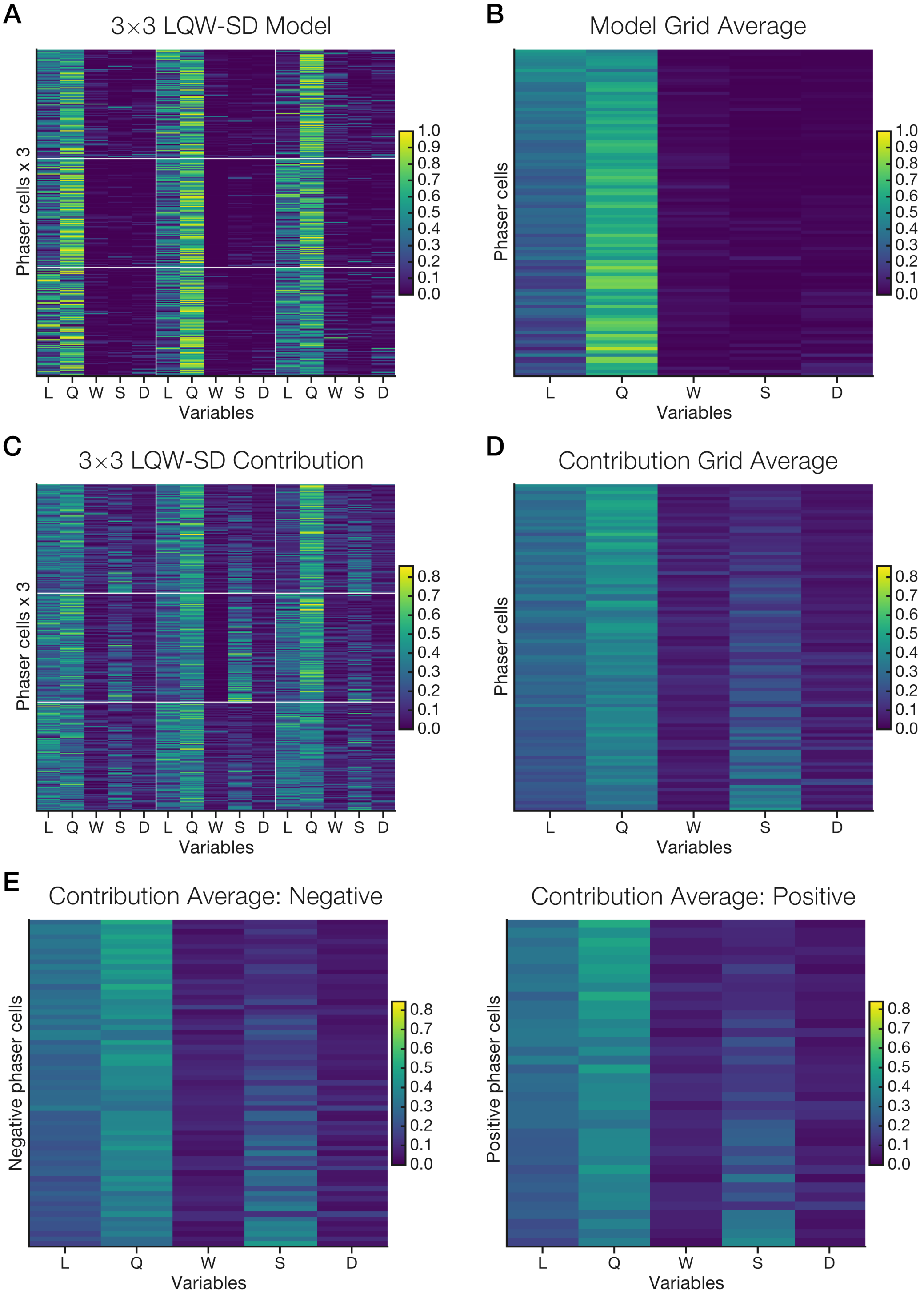

S6 Fig. *See legend on reverse.*

S6 Fig. GLM weights and contributions for every phaser cell recording. GLM weights (A+B) and maximal contributions (C-E) for phaser cell recordings are shown in pseudocolor matrix plots. For visualization, recordings are presented in the same order in every grid section and grid average according to the expected value of the cell's grid-averaged model weights to the left (toward L, i.e., more spatial) or right (toward D, i.e., more trajectory-related). To reveal model structure, each variable row in a grid section was sum-normalized and the paired grid plots (A+B, C+D) share color scales. (E) The contribution averages from (D) are displayed by phaser cell subtype: negative (left) and positive (right). The two subtypes demonstrated qualitatively similar inverse patterns of spatial (L, Q) vs. speed-related (S) contributions to firing.
