# Supplementary material for: Spatial synchronization codes from coupled rate-phase neurons
Source: PLoS Comput Biol. 2019 Jan 25;15(1):e1006741. doi: 10.1371/journal.pcbi.1006741 (PMC6364943; doi:10.1371/journal.pcbi.1006741)
Supplement: S7 Fig — An intrinsic bursting model (Eq (11); [51]) was tuned with constant input (Table 4) to fire doublet bursts (A) close to the reference theta frequency, 7.5 Hz. The deviation between the reference frequency and the resulting burst rate, 7.519 bursts/s, meant that the unit’s theta phase (B) slowly drifted (precessed) over time (gray line). To test whether this unit could be phase-synchronized by periodic stimulation, we simulated an instantaneous pulse (V ← V+ 15mV) every other theta cycle at theta peak (0 radians). This pulse-synchronized unit (B, orange line) monotonically delayed toward theta peak and then (around 5 s into the simulation) discontinuously jumped past theta peak before slowly precessing to just before the peak. This dynamic, of jumping forward and precessing back, repeated (around 9 s) and continued stereotypically. This sawtooth pattern encapsulated the model’s theta-synchronization dynamics. For simulations with phaser network input, we added a stochastic input current to this ‘target burster’ model (Eq (11)). We chose a noise level (Table 4) that preserved theta bursting (C, same as Fig 7C, inset) but caused its burst phase to randomly drift over a 30-s simulation (D, gray dots, 36 trials). With noise, the pulse stimulation was able to reproduce the sawtooth pattern of synchronization (D, orange line). (PDF) [file pcbi.1006741.s007.pdf]

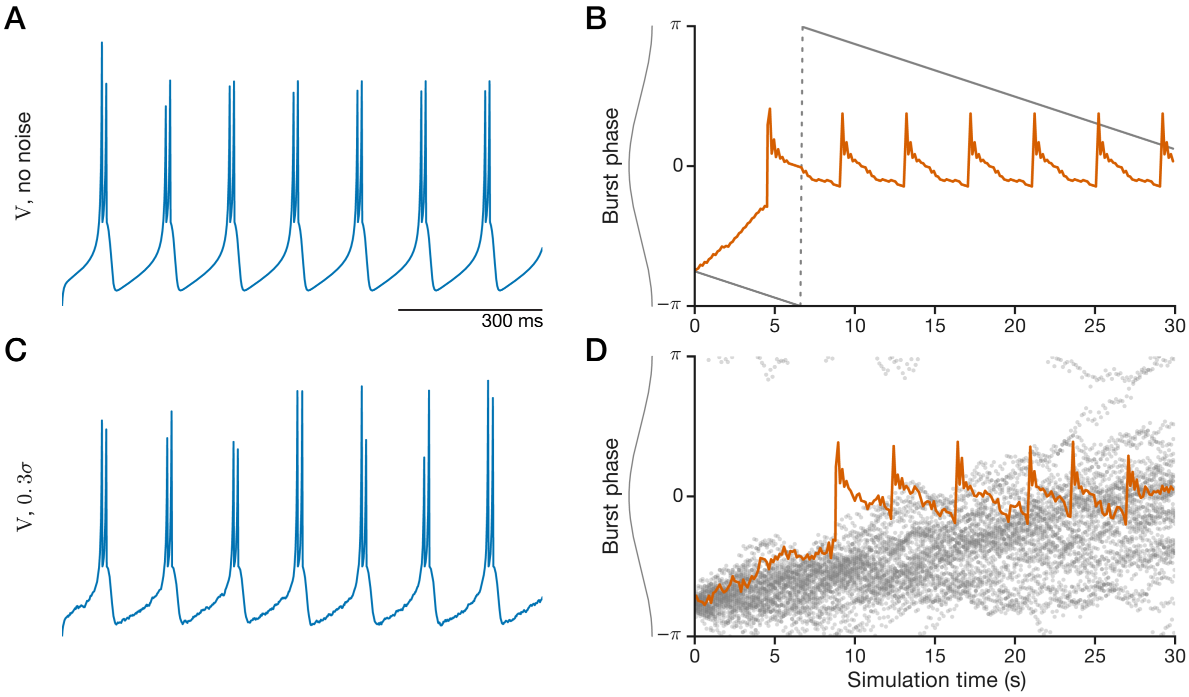

S7 Fig. Noisy theta-bursting target neuron model: Pulse synchronization. An intrinsic bursting model (Eq (11); [51]) was tuned with constant input (Table 4) to fire doublet bursts (A) close to the reference theta frequency, 7.5 Hz. The deviation between the reference frequency and the resulting burst rate, 7.519 bursts/s, meant that the unit's theta phase (B) slowly drifted (precessed) over time (gray line). To test whether this unit could be phase-synchronized by periodic stimulation, we simulated an instantaneous pulse ($V \leftarrow V + 15\,\mathrm{mV}$) every other theta cycle at theta peak (0 radians). This pulse-synchronized unit (B, orange line) monotonically delayed toward theta peak and then (around 5 s into the simulation) discontinuously jumped past theta peak before slowly precessing to just before the peak. This dynamic, of jumping forward and precessing back, repeated (around 9 s) and continued stereotypically. This sawtooth pattern encapsulated the model's theta-synchronization dynamics. For simulations with phaser network input, we added a stochastic input current to this 'target burster' model (Eq (11)). We chose a noise level (Table 4) that preserved theta bursting (C, same as Fig 7C, inset) but caused its burst phase to randomly drift over a 30-s simulation (D, gray dots, 36 trials). With noise, the pulse stimulation was able to reproduce the sawtooth pattern of synchronization (D, orange line).
