# Supplementary material for: Spatial synchronization codes from coupled rate-phase neurons
Source: PLoS Comput Biol. 2019 Jan 25;15(1):e1006741. doi: 10.1371/journal.pcbi.1006741 (PMC6364943; doi:10.1371/journal.pcbi.1006741)
Supplement: S8 Fig — We show additional 1-hr simulations of the 1D phaser-target network shown in Fig 7F+7G. (A) With the input gain from the phasers fixed (Table 4), simulations with 0.0σ, 0.1σ, 0.3σ, and 0.5σ noise levels demonstrated that the supervised modes of the artificial phase-code remained functional across different levels of noise. (B) With the noise level fixed at 0.3σ, the effect of zero phaser input gain (top left) can be compared to weaker (top right) and stronger (bottom right) levels of phaser input. Weak phaser input (top right) entrained the target burster, but the phase trajectories were extended due to the slower development of phase locking on approaches toward positions 0 or 1. (PDF) [file pcbi.1006741.s008.pdf]

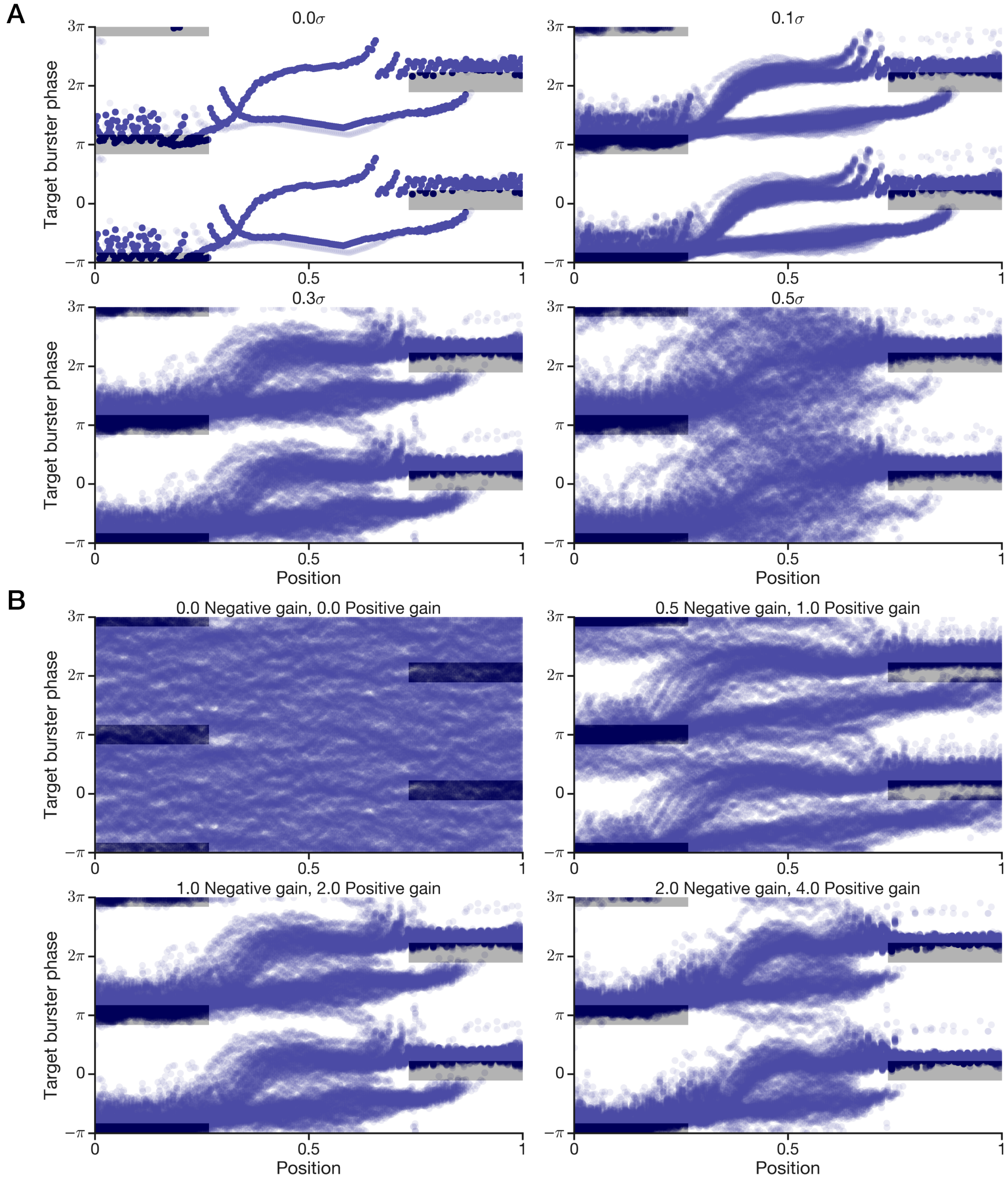

S8 Fig. *See legend on reverse.*

S8 Fig. 1D phaser-target entrainment across noise and phaser input levels. We show additional 1-hr simulations of the 1D phaser-target network shown in Fig 7F+G. (A) With the input gain from the phasers fixed (Table 4), simulations with 0.0σ, 0.1σ, 0.3σ, and 0.5σ noise levels demonstrated that the supervised modes of the artificial phase-code remained functional across different levels of noise. (B) With the noise level fixed at 0.3σ, the effect of zero phaser input gain (top left) can be compared to weaker (top right) and stronger (bottom right) levels of phaser input. Weak phaser input (top right) entrained the target burster, but the phase trajectories were extended due to the slower development of phase locking on approaches toward positions 0 or 1.
