# Supplementary material for: Spatial synchronization codes from coupled rate-phase neurons
Source: PLoS Comput Biol. 2019 Jan 25;15(1):e1006741. doi: 10.1371/journal.pcbi.1006741 (PMC6364943; doi:10.1371/journal.pcbi.1006741)
Supplement: S9 Fig — (A) Ratemap/phase-map pairs are shown for 50/1,000 negative phasers from the realistic 2D open-field simulations (Fig 8). The rate and phase response of each phaser was driven by a randomly sampled spatial function from the LQW generative input model (S10 Fig, panel A). In the phase maps, note that the phasers advanced from pre-theta-peak (green; see phase-vector color wheel at bottom) to theta-trough (pink) from low- to high-rate regions. Missing phase map pixels reflect insufficient numbers of nearby spikes for spatial averaging. (B) Ratemap/phase-map pairs are shown for 50/1,000 positive phasers. The rate and phase response of each phaser was driven by theta excitation and feedforward inhibition from a negative phaser with an LQW-generated spatial input (A). In the phase maps, note that the phasers delayed from theta peak (green) to halfway through the falling phase (blue/green; π/2 radians). Like the 1D model (Fig 6) and phaser cell recordings (Fig 4), the positive rate-phase coupling was weaker than the negative. (PDF) [file pcbi.1006741.s009.pdf]

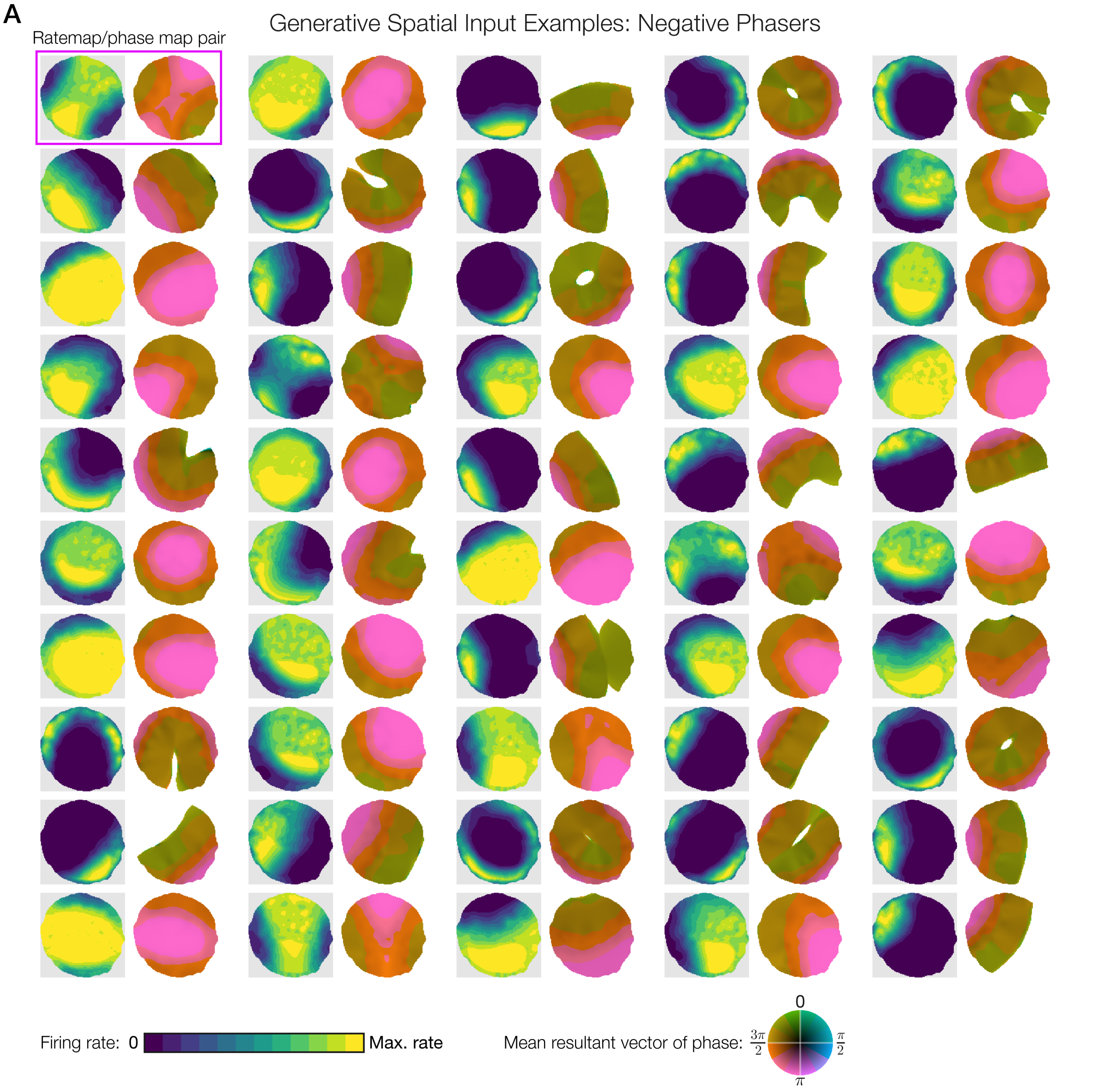

S9 Fig. Generative samples of model LQW-phasers in open-field simulations. (A) Ratemap/phase-map pairs are shown for 50/1,000 negative phasers from the realistic 2D open-field simulations (Fig 8). The rate and phase response of each phaser was driven by a randomly sampled spatial function from the LQW generative input model (S9 Fig, panel A). In the phase maps, note that the phasers advanced from pre-theta-peak (green; see phase-vector color wheel at bottom) to theta-trough (pink) from low- to high-rate regions. Missing phase map pixels reflect insufficient numbers of nearby spikes for spatial averaging. (B) *Continued on next page.*

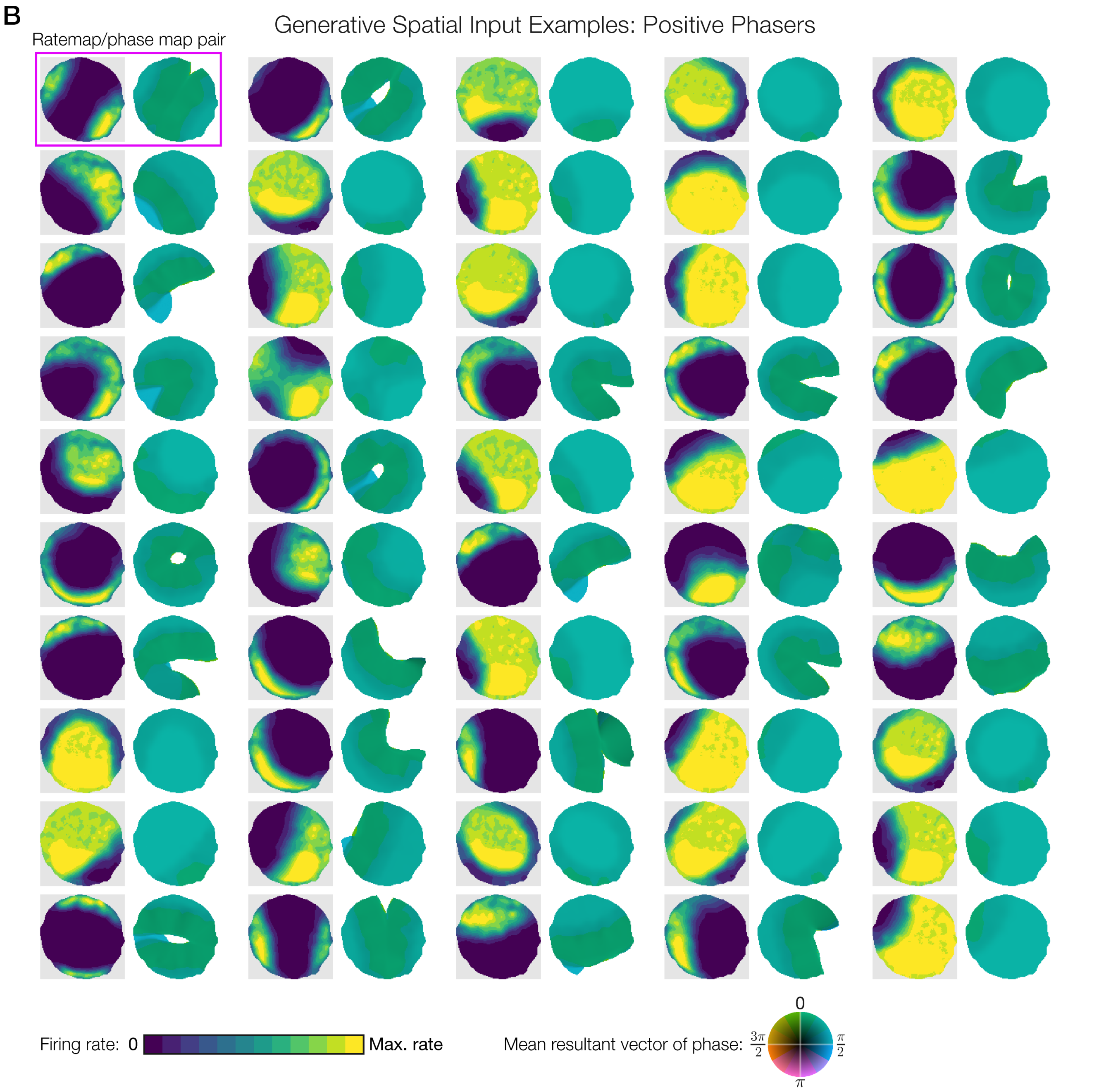

S9 Fig. (Continued). (B) Ratemap/phase-map pairs are shown for 50/1,000 positive phasers. The rate and phase response of each phaser was driven by theta excitation and feedforward inhibition from a negative phaser with an LQW-generated spatial input (A). In the phase maps, note that the phasers delayed from theta peak (green) to halfway through the falling phase (blue/green; $\pi/2$ radians). Like the 1D model (Fig 6) and phaser cell recordings (Fig 4), the positive rate-phase coupling was weaker than the negative.
