# Supplementary material for: Spatial synchronization codes from coupled rate-phase neurons
Source: PLoS Comput Biol. 2019 Jan 25;15(1):e1006741. doi: 10.1371/journal.pcbi.1006741 (PMC6364943; doi:10.1371/journal.pcbi.1006741)
Supplement: S10 Fig — Realistic 2D simulations of phasers and target neurons were simulated and the bursting activity of the target neurons was decoded to assess position-error correction (Methods). (A) The steps to sample spatial input functions from the generative model for negative phasers are illustrated (Methods). From left to right: Phaser cell recordings (examples from Fig 4A) were learned by the 1 × 1 LQW model (Eq (3)) and their linear predictor functions were normalized to [0, 1] with a sigmoid nonlinearity. To generate a novel spatial input, we randomly selected one of these normalized spatial functions, added 20% Gaussian noise to the LQW parameters, and randomly center-rotated the coordinate frame. (B) Target networks were simple collections of target burster units. The Ring collection of target bursters varied across phase offsets (orange); the Phase 1 and Phase 2 collections varied across preferred direction at opposing phase offsets (blue and green). (C) Normalized temporal autocorrelograms of decoding error for full-sized collections (64 units in each collection; 192 units for the combination of all collections). The correlation width indicates the timescale of error correction, which was quantified as the HWHM timescale in Fig 8H (Methods). (PDF) [file pcbi.1006741.s010.pdf]

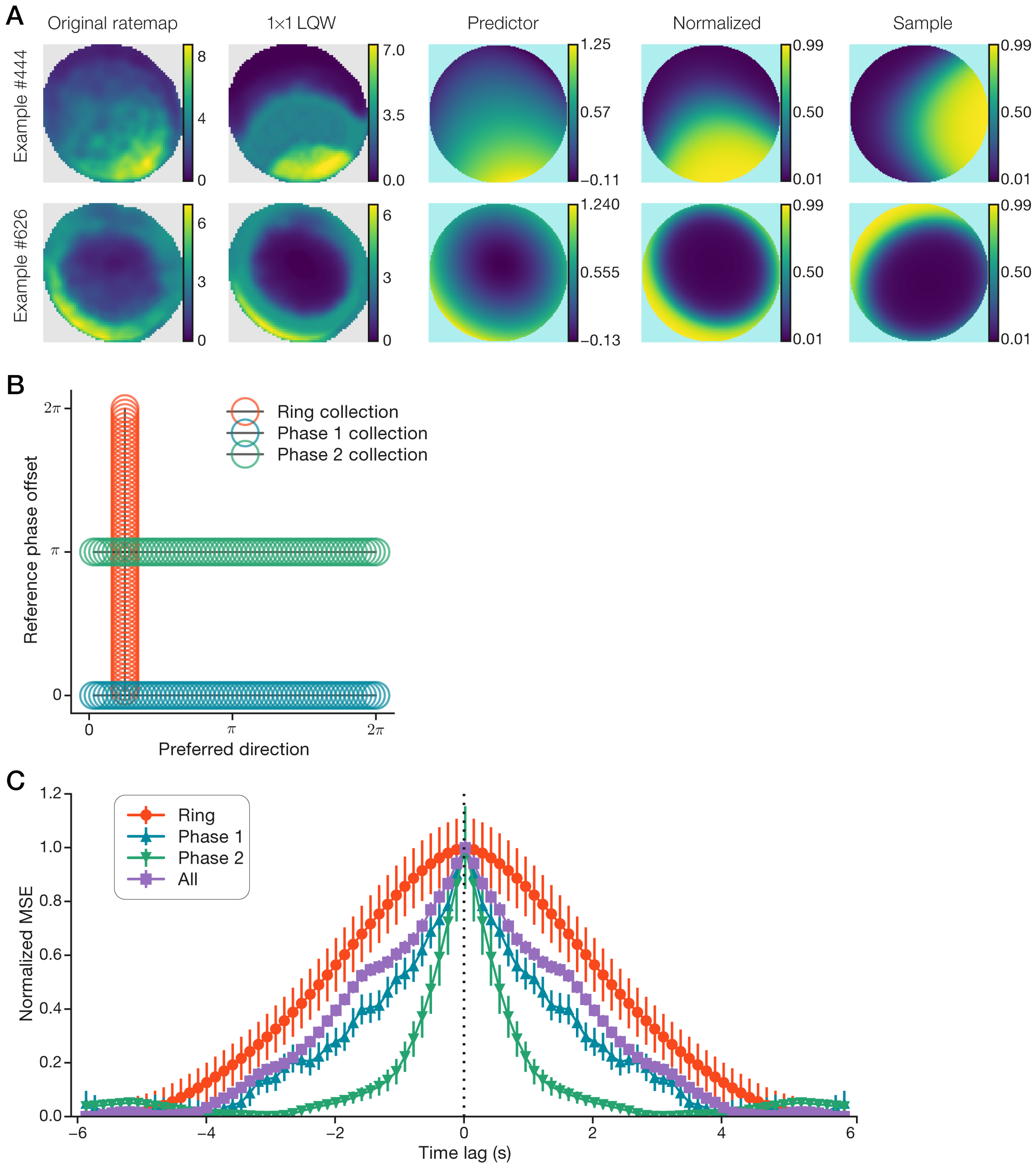

S10 Fig. *See legend on reverse.*

S10 Fig. Bayesian decoding of target burst phase from open-field simulations. Realistic 2D simulations of phasers and target neurons were simulated and the bursting activity of the target neurons was decoded to assess position-error correction (Methods). (A) The steps to sample spatial input functions from the generative model for negative phasers are illustrated (Methods). From left to right: Phaser cell recordings (examples from Fig 4A) were learned by the 1×1 LQW model (Eq (3)) and their linear predictor functions were normalized to $[0, 1]$ with a sigmoid nonlinearity. To generate a novel spatial input, we randomly selected one of these normalized spatial functions, added 20% Gaussian noise to the LQW parameters, and randomly center-rotated the coordinate frame. (B) Target networks were simple collections of target burster units. The Ring collection of target bursters varied across phase offsets (orange); the Phase 1 and Phase 2 collections varied across preferred direction at opposing phase offsets (blue and green). (C) Normalized temporal autocorrelograms of decoding error for full-sized collections (64 units in each collection; 192 units for the combination of all collections). The correlation width indicates the timescale of error correction, which was quantified as the HWHM timescale in Fig 8H (Methods).
